# Amphistomy: stomata patterning inferred from ^13^C content and leaf-side-specific deposition of epicuticular wax

**DOI:** 10.1093/aob/mcae082

**Published:** 2024-06-05

**Authors:** Balzhan Askanbayeva, Jitka Janová, Jiří Kubásek, Viktoria V Zeisler-Diehl, Lukas Schreiber, Christopher D Muir, Jiří Šantrůček

**Affiliations:** Department of Experimental Plant Biology, Faculty of Science, University of South Bohemia, Branišovská 31, 370 05 České Budějovice, Czech Republic; Department of Experimental Plant Biology, Faculty of Science, University of South Bohemia, Branišovská 31, 370 05 České Budějovice, Czech Republic; Department of Experimental Plant Biology, Faculty of Science, University of South Bohemia, Branišovská 31, 370 05 České Budějovice, Czech Republic; Institute of Cellular and Molecular Botany, University of Bonn, Kirschallee 1, 53115 Bonn, Germany; Institute of Cellular and Molecular Botany, University of Bonn, Kirschallee 1, 53115 Bonn, Germany; Department of Botany, University of Wisconsin, 143 Lincoln Drive, Madison, WI 53711, USA; Department of Experimental Plant Biology, Faculty of Science, University of South Bohemia, Branišovská 31, 370 05 České Budějovice, Czech Republic

**Keywords:** Amphistomy, leaf internal CO_2_ concentration, stomata, cuticle, epicuticular wax, adaxial, abaxial, carbon isotope, light, photosynthesis, *Brassica oleracea*, *Capsicum annuum*

## Abstract

**Background and Aims:**

The benefits and costs of amphistomy (AS) vs. hypostomy (HS) are not fully understood. Here, we quantify benefits of access of CO_2_ through stomata on the upper (adaxial) leaf surface, using ^13^C abundance in the adaxial and abaxial epicuticular wax. Additionally, a relationship between the distribution of stomata and epicuticular wax on the opposite leaf sides is studied.

**Methods:**

We suggest that the ^13^C content of long-chain aliphatic compounds of cuticular wax records the leaf internal CO_2_ concentration in chloroplasts adjacent to the adaxial and abaxial epidermes. This unique property stems from: (1) wax synthesis being located exclusively in epidermal cells; and (2) ongoing wax renewal over the whole leaf lifespan. Compound-specific and bulk wax ^13^C abundance (*δ*) was related to amphistomy level (ASL; as a fraction of adaxial in all stomata) of four AS and five HS species grown under various levels of irradiance. The isotopic polarity of epicuticular wax, i.e. the difference in abaxial and adaxial *δ* (*δ*_ab_ − *δ*_ad_), was used to calculate the leaf dorsiventral CO_2_ gradient. Leaf-side-specific epicuticular wax deposition (amphiwaxy level) was estimated and related to ASL.

**Key Results:**

In HS species, the CO_2_ concentration in the adaxial epidermis was lower than in the abaxial one, independently of light conditions. In AS leaves grown in high-light and low-light conditions, the isotopic polarity and CO_2_ gradient varied in parallel with ASL. The AS leaves grown in high-light conditions increased ASL compared with low light, and *δ*_ab_ − *δ*_ad_ approached near-zero values. Changes in ASL occurred concomitantly with changes in amphiwaxy level.

**Conclusions:**

Leaf wax isotopic polarity is a newly identified leaf trait, distinguishing between hypo- and amphistomatous species and indicating that increased ASL in sun-exposed AS leaves reduces the CO_2_ gradient across the leaf mesophyll. Stomata and epicuticular wax deposition follow similar leaf-side patterning.

## INTRODUCTION

Leaves, the primary organs of photosynthesis, solve several fundamental trade-offs, which include CO_2_ uptake through photosynthesis and water loss through transpiration. Certain leaf traits are considered crucial for plant adaptation to varying environmental conditions, especially light (Niinemets *et al.*, 2015; Poorter *et al.*, 2019). Leaf shape, size, anatomy (including vein pattern), stomatal density (SD; the number of stomata per unit leaf area), size and distribution are the main factors affecting rates of water and CO_2_ diffusion (Franks and Beerling, 2009; Adams and Terashima, 2018). The conductance for diffusion of CO_2_ into leaves and chloroplasts is regulated primarily by stomata and mesophyll (Haworth *et al.*, 2015; Flexas *et al.*, 2018; Lawson and Matthews, 2020).

It has been shown that the stomatal distribution between the upper (adaxial) and lower (abaxial) leaf surfaces is of adaptive importance in various environmental conditions (Mott *et al.*, 1982; Pathare *et al.*, 2020). Many herbaceous and some woody species are amphistomatous (AS), i.e. they have stomata on both leaf surfaces. In contrast, most tree species have stomata on only the lower leaf surface; such leaves are called hypostomatous (HS) (Willmer and Ficker, 1996). Epistomatic leaves, with stomata restricted to the upper leaf side, are typical for aquatic species with leaves floating on the water surface (Bjorn *et al.*, 2022; Liu *et al.*, 2023). Amphistomy, i.e. the presence of stomata on both leaf sides, is usually quantified in two different ways: (1) as the ratio of SD on the adaxial and the abaxial side (SD_ad_/SD_ab_), the so-called stomatal ratio (SR); or (2) as the fraction of adaxial stomata per total abundance of stomata on both leaf sides [SD_ad_/(SD_ab_ + SD_ad_)], termed the amphistomy level (ASL) here. The SR ranges from zero in HS species (SD_ad_ = 0) to infinity in epistomatous species with stomata on only the upper leaf side (SD_ab_ = 0), whereas ASL scales, more conveniently, between zero and one for the same extremes and is 0.5 when adaxial and abaxial sides bear the same number of stomata (SD_ab_ = SD_ad_). For this reason, we use ASL in this study. The ASL can change in a single species in response to irradiance and other environmental variables and depends on plant growth strategy, for example on root-to-shoot ratio (Mott *et al.*, 1982; Muir, 2015; Fontana *et al.*, 2017).

Amphistomy is associated with functional benefits, i.e. an increase in CO_2_ flux into and inside the leaf, hence higher photosynthetic rate (Parkhurst, 1994; Muir *et al.*, 2014; Pathare *et al.*, 2020). In ontogeny, plants have two options for producing leaves with greater maximal stomatal conductance: (1) increase stomatal density and/or size on the lower leaf surface; or (2) develop additional stomata on the upper leaf surface, i.e. increase ASL (Mott *et al.*, 1982). Low conductance for CO_2_ can limit photosynthetic assimilation, especially in leaves exposed to high light in open habitats. An increased proportion of adaxial stomata appears a feasible way to adapt to these conditions, especially when it is coupled with an increase in the total number of stomata per unit projected leaf surface area. Mott and Michaelson (1991) showed that *Ambrosia cordifolia* leaves grown in high-light conditions were AS, but leaves developed under low-light conditions were HS, and the acclimation to high light involved an increase in leaf thickness. The proportionality between leaf thickness and AS (Parkhurst, 1978) indicates the genomic and environmentally based alleviation of unfavourably long diffusional pathways inside thick leaves (Mott *et al.*, 1982; Mott and Michaelson, 1991; Drake *et al.*, 2019). Selection for easier CO_2_ access seems to be a strong driver in AS evolution (Muir, 2015). On the contrary, AS also has disadvantages, viz. a higher probability of bacteria and fungi penetrating through stomata on the upper leaf surface and increased water loss (McKown *et al.*, 2014). The leaf hydraulic system can also place significant constraint on ASL and, vice versa, AS can bring benefits in higher efficiency of leaf hydraulics use (Drake *et al.*, 2019; Richardson *et al.*, 2020).

Amphistomy is not exclusive to a specific plant life form, perennial or annual, herb or tree. Each of them includes species without adaxial stomata; nevertheless, AS occurs more widely in annual, fast-growing herbs, in trees with isobilateral leaves or in graminoids with vertically oriented leaves (Muir, 2015; Pathare *et al.*, 2020). It has been shown that woody species with AS leaves are more abundant in open vegetation and dry climates (Cooper and Cass, 2003; Jordan *et al.*, 2014). So far, it is not well understood what level of AS is optimal for a given species and ambient conditions, mainly light environment. Should AS develop in order to open the additional CO_2_ access and alleviate its shortage in mesophyll cells? In the positive case, a reduction of CO_2_ concentration ([CO_2_]) gradient across the leaf might be expected, and the gradient could approach zero at the optimal level of AS. We tested this hypothesis here.

Plant discrimination against ^13^CO_2_ in photosynthesis (expressed as Δ^13^C) offers an efficient method for elucidating various parameters of CO_2_ uptake, especially for estimation of leaf intercellular [CO_2_] (*c*_i_), which is directly proportional to Δ^13^C (Farquhar *et al.*, 1982). It is widely accepted that the [CO_2_] in chloroplasts is proportional to the depletion of ^13^C in primary assimilates relative to the ambient atmosphere. Muir *et al.* (2014) found that the SR was correlated with carbon isotope discrimination in wild tomato species. Leaves with higher SR discriminated against ^13^C more strongly, i.e. they were depleted in ^13^C compared with the low-SR leaves, indicating that AS increases [CO_2_] in chloroplasts by facilitating stomatal and leaf internal CO_2_ diffusion. Here, we extend this approach to leaf-side-specific discrimination, assuming that synthesis of lipids in the epidermis and their deposition as epicuticular wax (EW) specifically ‘fingerprints’ the [CO_2_] in the adjacent mesophyll chloroplasts and plastids for the adaxial and abaxial epidermis, respectively. This assumption is based on the knowledge that the synthesis of fatty acids in plastids and subsequent synthesis of lipids in endoplasmic reticulum is a cell-autonomous process (fatty acids and glycerolipids are not transported between cells at an extensive scale) and that only epidermal cells possess the exclusive property to export lipids extracellularly, i.e. to the cuticle (Somerville *et al.*, 2000). However, triose phosphates, as the primary assimilates in C_3_ plants, can undergo further post-photosynthetic ^13^C fractionation, which, depending on which further metabolic processes are involved, affects the chloroplastic [CO_2_] imprint more or less strongly (Hobbie and Werner, 2004; Badeck *et al.*, 2005). The synthesis of long-chain aliphatic wax compounds starts with synthesis of fatty acids with carbon chain lengths of 16 and 18 in mesophyll chloroplasts and plastids of epidermal cells from pyruvate and continues by elongation in the endoplasmic reticulum (Somerville *et al.*, 2000; Kunst and Samuels, 2009; Lee and Suh, 2022). The kinetic isotope effect during catalytic activity of the pyruvate dehydrogenase complex is responsible for the commonly observed ^13^C depletion of lipids compared with the primary assimilates (trioses) or plant dry mass (mainly cellulose) (DeNiro and Epstein, 1977; Melzer and Schmidt, 1987; Hayes, 2001). However, this post-photosynthetic discrimination of lipids is, presumably, not a leaf-side-specific process. More evidence supporting the assumption on imprinting of [CO_2_] in plastids into the cuticular lipids is mentioned in the Discussion.

The epidermal cell autonomy in lipid synthesis leads us to expect that the adaxial and abaxial cuticular waxes should differ in ^13^C content according to the [CO_2_] in adjacent chloroplasts (*c*_c_). Given that *c*_c_ is affected by the presence or absence of stomata in the epidermis, the ASL should also impact on the leaf-side-specific *c*_c_ and ^13^C abundance in the waxes. Recently, we suggested a technique that allowed us to assess the relative difference of [CO_2_] between mesophyll cells adjacent to the adaxial and abaxial leaf sides, in HS species (Santrucek *et al.*, 2019). Here, we aimed to test whether the ASL affects the gradient of [CO_2_] across the leaf (note that ‘gradient’ here and below refers to the difference in [CO_2_] between the epidermes, not to the change in [CO_2_] per unit of leaf thickness) or, vice versa, whether the frequency of stomata on the upper leaf surface is linked to the availability of CO_2_ in the mesophyll underlying the upper epidermis. First, we compare the leaf-side-specific ^13^C content in wax from several HS and AS species. Second, we compare, in a more detailed study, two AS species [pepper (*Capsicum annuum* L.) and broccoli (*Brassica oleracea* L.)], with different ASLs modulated by growth in low or high light and by leaf insertion level. Together with the leaf-side-specific ^13^C content, we estimate the quantity of EW deposited on the adaxial and abaxial sides and its relationship to ASL.

## MATERIALS AND METHODS

### Plant material and growth conditions

#### Pilot experiment

In a pilot experiment, we collected mature leaves of five HS and four AS species from plants grown in natural conditions (HS: *Prunus laurocerasus* and *Eucalyptus diversicolor*; AS: *Araucaria bidwillii*) or cultivated in a glasshouse (HS: *Zamioculcas zamiifolia*, *Schefflera arboricola* and *Euonymus japonicus*) or in growth chambers (AS: *Brassica oleracea*, *Capsicum annuum* and *Plantago major*). The species grown in growth chambers were exposed to two contrasting light conditions (see the next paragraph). Leaves of *Eucalyptus diversicolor* (karri tree) were collected from the top and bottom parts of the crown of a mature tree growing in Western Australia (Gloucester National Park). Twigs with needles of *A. bidwillii* (bunya pine) were obtained from the top of three mature trees and of three trees in the understorey in Bunya Mountains National Park, Queensland, Australia (see photographs in Supplementary Data Fig. S1). In all nine species, we sampled EW from, and determined stomatal density on, both leaf sides. We analysed carbon isotope composition (expressed as *δ*^13^C) of four to seven major wax components in each species (see below for details).

#### Detailed experiment with pepper and broccoli

In a separate experiment, two fast-growing AS plants, pepper (paprika) and broccoli, were grown and analysed in a more detailed way. The pepper (*C. annuum*) cultivar *Slovakia* (Semo, Czech Republic) and the broccoli (*B. oleracea*) cultivar *Limba* (Moravo Seed, Czech Republic) were grown from seeds in pots filled with soil (commercial garden mixture B, Raselina Sobeslav, Czech Republic). The plants were watered two or three times a week, alternatively with tap water and half-strength nutrient solution (Kristalon Gold, Agro CS, Czech Republic). Five experimental runs were organized. In two runs (February–March 2020 and July–September 2020), the plants were grown in a Fitotron growth chamber (Sanyo, UK), and for the remaining three runs (November–December 2020, January–March 2021 and September 2021–March 2022) we used a Percival growth chamber (Percival Scientific, Inc., USA). In each run, plants were grown under two levels of irradiance, high light (HL; 450 ± 50 μmol m^−2^ s^−1^ photosynthetic photon flux density) and low light (LL; 100 ± 20 μmol m^−2^ s^−1^ photosynthetic photon flux density), at day/night temperatures of 25/17 °C, 16 h photoperiod and free atmospheric [CO_2_] of 360–420 µmol mol^−1^. The spectral compositions of photosynthetically active radiation were identical in HL and LL but differed slightly between Fitotron and Percival chambers (Supplementary Data Fig. S2). Fully developed leaves of 8- to 13-week-old plants were investigated (Supplementary Data Fig. S3). In the first four runs, we evaluated the effect of light as a single factor affecting leaf anatomy, stomata patterning and ^13^C content in bulk leaf mass and wax. In the fifth run, organized separately for pepper and broccoli, leaf insertion level (leaf age) and wax components were included, additionally to the factor of light. Nine repetitions for each old (low insertion level), mature (middle insertion level) and young (upper insertion level) leaves were obtained in each light environment.

### Leaf anatomy

Leaf thickness and mesophyll cell density (packing) can substantially affect CO_2_ access and concentration in chloroplasts. Therefore, we measured the thickness of leaves grown in HL and LL conditions on transverse leaf sections under an optical microscope. Leaf samples were fixed with 2.5 % glutaraldehyde in 0.1 m phosphate buffer; specimens were dehydrated through a series of graded concentrations of acetone, embedded in epoxy resin and stained with Toluidine Blue. Semi-thin sections (400 nm) were cut using an ultramicrotome (Leica EM UC6, Leica Mikrosysteme, Vienna, Austria). A light microscope (Olympus Bx61) with ×20 magnification and a digital camera (Canon EOS 750D) were used to photograph leaf transects. The cross-sections were analysed with ImageJ software. Five transects were used to measure leaf thickness.

### Estimation of SD and ASL

Stomatal density was determined with ImageJ software on photographs of nail polish imprints obtained directly from both leaf surfaces. A light microscope (Olympus BX61) with a ×50 magnification objective and a digital camera (Canon EOS 750D) were used to photograph a leaf surface area of 0.130 mm^2^. Three to five areas without leaf veins were selected randomly per leaf side. Ten to fourteen leaves were evaluated per species and growth light treatment in the first four experimental runs; nine leaves per insertion level, light and species were analysed in the fifth run. The results were expressed as number of stomata per millimetre squared [stomatal density (SD)] of abaxial and adaxial leaf sides. The ASL was calculated as follows: ASL = SD_ad_/(SD_ad_ + SD_ab_).

### Wax sampling with collodion

The procedure used for obtaining EW from the leaf surface was as described in detail by Zeisler and Schreiber (2016). Briefly, collodion (solution of nitrocellulose in ethanol/diethyl ether; Merck, Germany) was applied with a brush to the upper and lower sides of the leaf and peeled off after drying (~1 min), stripping the surface wax with it. Then, the collodion strip with the adherent EW layer was put into glass scintillation vials with 2–4 mL of chloroform or *n*-hexane (in run 5), closed and extracted on a roller (DLAB, China) overnight. The nitrocellulose peel was removed, solvent evaporated and the amount of harvested wax estimated gravimetrically with a microbalance (Mettler Toledo MT5, Italy). Finally, the wax was redissolved in chloroform or *n*-hexane, and the solution of wax, after concentration by partial evaporation, was moved to an open tin capsule and evaporated further, rendering an amount of wax (70–100 µg) suitable for elementar analyser-isotope ratio mass spectrometer analysis, or used directly for compound-specific carbon isotope analyses by gas chromatography coupled with stable isotope ratio mass spectrometry (GC-IRMS). Fresh leaves were used in the wax collection, except for *Eucalyptus diversicolor* and *A. bidwillii*, which were desiccated in dry air at ambient temperature after sampling, transferred to the Czech Republic, and their wax was collected from dry leaves as described above. In experiment with pepper and broccoli, one or two (old), three (mature) or four (young) leaves, according to the insertion-level category, yielded wax for one sample; three samples per insertion and three insertions per plant were analysed. The leaves used in wax sampling were scanned and their area determined by ImageJ (Schneider *et al.*, 2012).

### Carbon isotope analyses in bulk material

The relative abundance of ^13^C over ^12^C (*δ*^13^C) in the bulk leaf dry matter and in the extracted wax was analysed in pepper and broccoli samples. Two to three leaf discs (diameter 2 cm or equivalent leaf mass) per sample were punched from the leaf, dried, ground to a fine powder with a ball mill (Retsch MM200, Haan, Germany) and packed in a tin capsule. The EW extracts previously dissolved in chloroform and evaporated in tin capsules were also packed. Epicuticular wax harvested from one leaf side was used for preparing one sample. Tin capsules were oxidized in a stream of pure oxygen by flash combustion at 950 °C in the reactor of an elemental analyser (Flash 2000, Thermo Fisher Scientific, Bremen, Germany) connected via continuous flow to a stable isotope ratio mass spectrometer (IRMS) (Delta plus XL, ThermoFinnigan, Bremen, Germany), where the ^13^C:^12^C ratio (*R*) was detected in the CO_2_ produced by combustion. The ^13^C content (*δ*^13^C) was calculated as the difference in relative abundance of ^13^C normalized to that in the VPDB (Vienna Pee Dee Belemnite) standard as *δ*^13^C = (*R*_sample_ − *R*_VPDB_)/*R*_VPDB_, where *R* stands for the ratio of concentrations of carbon isotopes ([^13^C]/[^12^C]). The standard deviation of *δ*^13^C determination in standard samples did not exceed 0.1 ‰.

### Compound-specific carbon isotope composition of EW

Information on availability of CO_2_ at the opposite leaf sides obtained from measurements of the isotopic composition of bulk EW might be biased owing to different isotopic signals of individual wax components. Therefore, we also used GC-IRMS (Trace 1310, Delta V Advantage, ThermoFisher, Germany) to determine *δ*^13^C of two to seven major compounds in EW. Chloroform or *n*-hexane extracts of wax were concentrated into 2 mL chromatographic vials to obtain compound concentrations in the range of 5–20 µg mL^−1^. A subsample of 1–2 µL was injected, using a split/splitless injector, into a deactivated glass liner with an internal diameter of 4 mm. The column Restek Rxi-5MS-Syl (30 m × 0.25 mm × 0.25 µm film thickness) was used with a flow rate of 1.5 mL min^−1^ of helium as a carrier gas. The injection (at 300 °C) was splitless for 1.5 min at 50 °C, then split into 100 mL min^−1^ for 1 min and 5 mL min^−1^ for the rest. The oven temperature programme was as follows: 50 °C during the injection and for the next 2 min, increase at 40 °C min^−1^ to 200 °C, further increase at 4 °C min^−1^ to 310 °C, and isothermal at 310 °C for the rest of the analysis. The internal standard *n*-tetracosane (C24 alkane) calibrated for *δ*^13^C against VPDB was added to the samples at a concentration of 10 or 20 µg mL^−1^ to quantify the compounds and check for isotopic offsets. Chloroform extracts of adaxial and abaxial EW from leaves of nine species (five HS and four AS) were analysed in the pilot experiment. In detailed experiments with broccoli and pepper, *n*-hexane solutions of adaxial and abaxial wax were analysed (108 samples per species). In order to obtain the qualitative and quantitative wax composition of *Capsicum* and *Brassica*, wax sampled with collodium was also analysed using gas chromatography coupled to flame ionization detection and gas chromatography coupled to mass spectrometry. Methodological details are given in the Supplementary Data (Fig. S4).

### CO_2_ drawdown across the leaf

Estimation of the difference in [CO_2_] between the abaxial and adaxial leaf sides was based on the carbon isotope composition of the leaf-side-specific EW, *δ*_ad_ and *δ*_ab_, and of the leaf dry mass, *δ*_l_. The relative [CO_2_] difference, i.e. the drawdown between the adaxial and abaxial leaf sides, was calculated as (Santrucek *et al.*, 2019):


$$\begin{array}{l} \frac{c_{ad}-c_{ab}}{c_{ab}}=-{\left(1+\frac{\delta_{l}-\delta_{b}+a}{\delta_{ab}-\delta_{ad}}\right)}^{-1}, \end{array}$$ (1)

where *c*_ad_ and *c*_ab_ denote [CO_2_] in subepidermal cells on the adaxial and abaxial leaf sides, respectively, *a* is the isotopic effect imposed by CO_2_ diffusion in still air (4.4 ‰), and the indices l, b, ad and ab of isotopic composition *δ* indicate bulk leaf mass, CO_2_ isotopically modified by diffusion through the stoma, adaxial EW and abaxial EW, respectively. We assumed that *δ*_b_ was close to −9.6 ‰. This value reflects the partial isotopic effect of diffusion of ambient CO_2_ with *δ*_a_ = −8.5 ‰ through the stomatal pore of a typical C_3_ plant having a ratio of substomatal to ambient [CO_2_] of *c*_i_:*c*_a_ = 0.75 [*δ*_b_=*δ*_a_ − *a*(1 − *c*_i_/*c*_a_) = −9.6 ‰]. An error in δ_b_ in the range of 1 ‰ caused by this simplification would produce an error in *c*_ad_:*c*_ab_ of ≤1 % (for more details, see Santrucek *et al.*, 2019). The CO_2_ drawdown is typically negative in HS leaves (*c*_ad_ < *c*_ab_). Depending on environmental conditions, photosynthetic capacity, leaf (photo)respiration and stomatal conductance, each of them being leaf-side specific, the drawdown in AS leaves can be negative, positive or close to zero.

### Potential levels of amphistomy and of the side-specific wax deposition

Our data indicated a proportional relationship between stomatal densities on both leaf sides. We used linear approximation of the SD_ad_ vs. SD_ab_ relationship for prediction of the asymptotic value of ASL at high values of SD_ad_ as follows. The SD_ad_ can be related to SD_ab_ by linear regression with the slope *k* and intercept *a*: SD_ad_ = *k* × SD_ab_ + *a*. It can be shown that ASL can be expressed in terms of *k* and *a* as:


$$\begin{array}{l} ASL=\frac{k}{k+1-\left(a/SD_{ad}\;\right)}\;\;\;\;\;\;\;\;\;. \end{array}$$ (2)

It follows from this expression that ASL limits to *k*/(*k* + 1) for values of SD_ad_ much higher than *a*. The asymptotic value of ASL determines the potential ASL (ASL_pot_ = *k*/*k* + 1). It is worth mentioning that *a* near zero (regression line intercepts the origin of axes) indicates that ASL does not show any dependence or shows only a mild dependence on the factor (e.g. light) causing variation in SD and, vice versa, an intercept *a* far from zero shows that ASL depends on the treatment producing the SD_ad_~SD_ab_ linear relationship.

In analogy to ASL and ASL_pot_, we evaluated the fraction of EW deposited on adaxial leaf side and denote it the amphiwaxy level (AWL): AWL = *W*_ad_/(*W*_ad_ + *W*_ab_), where *W* stands for EW coverage, in units of mass per leaf surface area (e.g. nanograms per centimetre squared). Given that the logarithmic transformation of *W* often produced a higher coefficient of determination (*R*^2^) of the linear plot of *W*_ad_ against *W*_ab_, we used the logarithmic form to calculate AWL and AWL_pot_, by analogy to eqn (2).

### Statistical evaluation

Data were analysed with the program SigmaPlot v.13.0 (Systat Co.) and are presented as the mean ± s.d. One-way ANOVA with Tukey’s test for *post hoc* comparisons, Student’s unpaired *t-*test or the Mann–Whitney rank sum test were used for comparisons of the ASL between HL and LL treatments and of the isotopic composition of leaf discs between HL and LL treatments in both species. Two-way ANOVA was used for testing the significance of the effect of growth light, age (leaf insertion) and their interaction on (*δ*_ab_ − *δ*_ad_), ASL and AWL and wax coverage. The Holm–Sidak test was applied in multiple comparison procedures.

We adopted a Bayesian path analytical approach to test for an association between ASL and (*δ*_ab_ − *δ*_ad_). We considered two alternative paths for association: a direct negative correlation between ASL and (*δ*_ab_ − *δ*_ad_) or, alternatively, an indirect negative association between variables mediated by effects of leaf age and/or light treatment. For each species, we estimated: (1) fixed effects of ASL (direct effect), leaf age and light treatment on (*δ*_ab_ − *δ*_ad_), with a random effect of replicate to account for repeated measures within individual; and (2) fixed effects of leaf age and light treatment on ASL, with a random effect of replicate. This approach enables us to tease apart the direct effect of ASL on (*δ*_ab_ − *δ*_ad_) from indirect association mediated by other variables. We fitted the model in *Stan* v.2.33.1 (Stan_Development_Team, 2023) using the R package *brms* v.2.20.4 (Bürkner, 2017, 2018) with *cmdstanr* v.0.6.1 (Gabry *et al.*, 2023) backend. We sampled the posterior distribution for 1.6 × 10^4^ iterations using Hamiltonian Monte Carlo after 1.6 × 10^4^ warm-up iterations, with a thinning interval of eight, on four chains until convergence ($\hat{R}\approx 1$). We calculated parameter estimates and confidence intervals from posterior samples using the median and 95 % quantiles, respectively. We repeated the same analysis to test for an association between ASL and the CO_2_ gradient across the leaf, $\left[\left(c_{ad}-c_{ab}\right)/c_{ab}\;\right]$. Scripts for running models are available on GitHub (https://github.com/cdmuir/amphiwaxy).

## RESULTS

### Overview of compound-specific isotopic composition of wax across HS and AS species

**Fig. 1.**
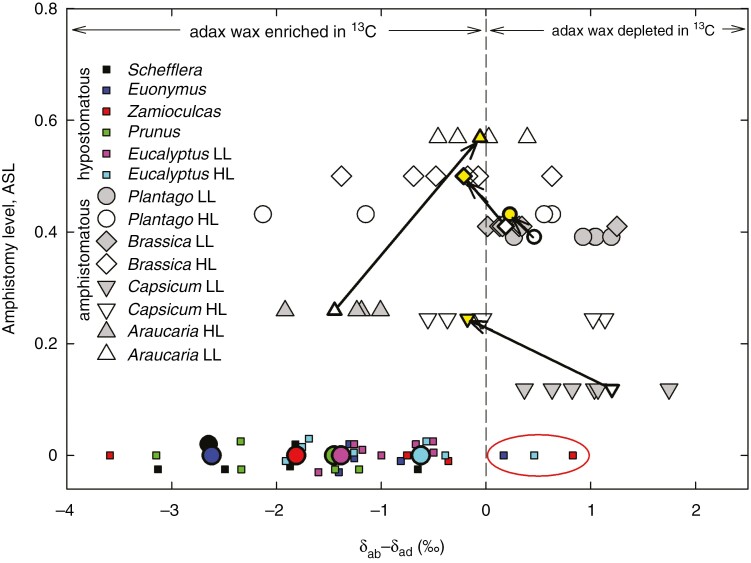
Differences in ^13^C abundance between abaxial and adaxial leaf epicuticular wax (*δ*_ab_ − *δ*_ad_) as related to amphistomy level (ASL) for five hypostomatous (HS) and four amphistomatous (AS) plant species. Wax compounds from HS species are marked with species-specific coloured symbols. Squares represent different compounds dominant in epicuticular wax of the given species; the six coloured circles show values of (*δ*ʹ_ab_ − *δ*ʹ_ad_), where *δ*ʹ is *δ* weighted by the abundance of individual compounds. All HS species have an ASL of zero; therefore, some of the 33 symbols (26 compounds) were slightly shifted up or down in order to make them visible. In AS species, the grey symbols indicate low-light (LL; shaded) and open symbols high-light growth conditions (HL; sun exposed). The continuous lines with arrows connect mean values of (*δ*_ab_ − *δ*_ad_) from LL towards HL, weighted by the abundance of the individual wax components. For abundance of individual compounds, their *δ*_ab_ and *δ*_ad_ and weighted means, see Supplementary Data Table S1. The enrichment of adaxial wax in ^13^C (*δ*_ab_ − *δ*_ad_ < 0) indicates depression of [CO_2_] in the adaxial compared with the abaxial chloroplasts, whereas ^13^C depletion of adaxial wax (*δ*_ab_ − *δ*_ad_ > 0) points to the opposite [CO_2_] gradient across the leaf. An ASL value of 0.5 indicates identical stomatal density on both leaf sides.

Altogether, 26 different compounds (four to seven per species) with the highest abundance in EW collected from both leaf sides were analysed in five HS species. Seven compounds in *Eucalyptus diversicolor* wax were analysed twice, from both shaded and sun-exposed leaves. Of those 33 instances, 30 were enriched in ^13^C at the adaxial compared with the abaxial leaf side, which indicates depression of [CO_2_] in plastids at the adaxial astomatous compared with the abaxial stomatous leaf side; the three exceptions are marked with a red oval in Fig. 1. In contrast, there was no clear pattern to the distribution of *δ*^13^C differences between the leaf sides (*δ*_ab_ − *δ*_ad_) in AS species, although there was some dependence on light. The waxes of LL leaves were usually depleted in ^13^C at the adaxial compared with the abaxial side [(*δ*_ab_ − *δ*_ad_) > 0], with the exception of *A. bidwillii* (Fig. 1). In contrast, adaxial wax was almost identical in ^13^C content to abaxial wax [(*δ*_ab_ − *δ*_ad_) ≈ 0] of leaves grown in HL (yellow symbols for AS species in Fig. 1). This pattern applied to the amount-weighted mean *δ*^13^C of the major wax components, although data for individual compounds were scattered. Relative abundances and *δ*^13^C of the major wax components identified by their retention indices are shown in Supplementary Data Table S1. Taken together, AS and HS leaves typically exhibit inverse polarity in abundance of ^13^C in EW: the upper (adaxial) side of HS leaves is usually enriched in ^13^C against the lower (abaxial) side, whereas in AS leaves the adaxial side is usually depleted.

### Detailed study of two AS species

The following subsections address the leaf traits of AS species only, especially pepper and broccoli, which are herbaceous fast-growing crops suitable for repeated independent cultivation in growth boxes and having different sensitivity of ASL to light. Both species also differ remarkably in the quality and quantity of EW (Supplementary Data Fig. S4). The data were averaged over the four independent experimental runs; the fifth run is evaluated separately.

### Leaf thickness, stomatal density and ASL; effect of light

**Fig. 2.**
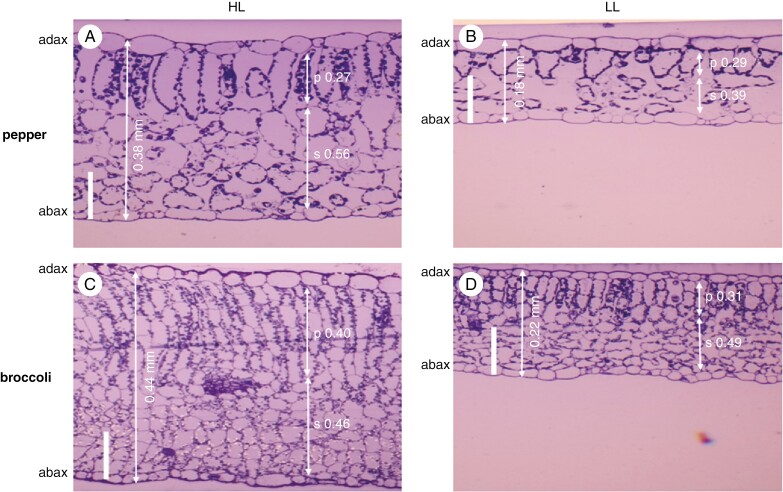
Light micrographs of leaf transverse sections of pepper and broccoli leaves grown at contrasting irradiance levels. (A) Pepper grown in high light [HL, 450 ± 50 µmol m^−2^ s^−1^; leaf thickness 0.38 ± 0.03 mm (mean ± s.d., *n* = 5)]. (B) Pepper grown in low light (LL, 100 ± 20 µmol m^−2^ s^−1^; leaf thickness 0.18 ± 0.02 mm). (C) Broccoli grown in HL (leaf thickness 0.44 ± 0.03 mm). (D) Broccoli grown in LL (leaf thickness 0.22 ± 0.02 mm). Abbreviations: p and s indicate fractions of palisade and spongy parenchyma tissue in the average total leaf thickness (in millimetres); scale bars: 0.1 mm.

The effects of contrasting light on the anatomy of pepper and broccoli leaves are shown in Fig. 2. As expected, leaves grown in HL conditions are about twice as thick as leaves grown in LL. The HL leaves have longer palisade cells in pepper and an additional layer of palisade cells in broccoli. Spongy mesophyll cells in LL leaves are not packed as tightly together as in HL leaves in both species (Fig. 2).

**Fig. 3.**
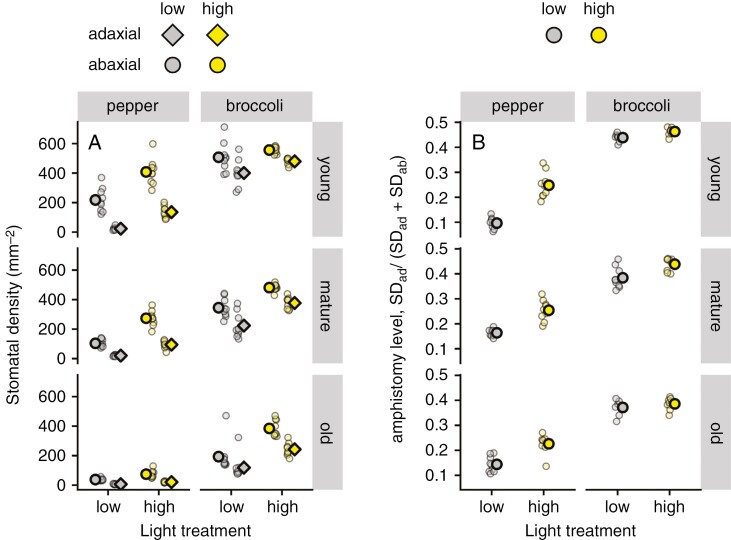
Stomatal density (SD) and amphistomy level of pepper and broccoli leaves of different age grown in high-light (450 ± 50 μmol m^−2^ s^−1^) and low-light treatments (100 ± 20 μmol m^−2^ s^−1^). The SD on adaxial and abaxial leaf sides (A) and amphistomy level (B) were estimated in young, mature and old leaves. Means (bold symbols) and individual measurements (*n* = 9) are shown. Data were obtained in the fifth experimental run. Pooled data from runs 1–4 are shown in Supplementary Data Fig. S6.

Stomatal density in both pepper and broccoli was affected by light exposition. As expected, total SD (SD_ad_ + SD_ab_) and the leaf-side-specific SD increased under elevated light intensity (Fig. 3A). However, SD on the adaxial leaf side increased more strongly (3.8-fold) than SD on the abaxial side (1.7-fold) in pepper, whereas in broccoli both leaf sides responded to higher light with approximately similar sensitivity (1.3-fold rise on both sides). The ASL in both species was <0.5 (i.e. more stomata on the lower than upper leaf side; Fig. 3B); however, the ASL response to light intensity differed between species (*F* = 46.6, *P* < 0.001 for plant species vs. light). Shading turned pepper towards hypostomy (*t* = 12.765, d.f. = 23, *P* < 0.001), whereas in broccoli the light intensity had no statistically significant effect on ASL (*t* = 1.939, d.f. = 18, *P* = 0.068) (see Fig. 3 for results from the fifth run and Supplementary Data Fig. S5 for runs 1–4).

### Abundance of ^13^C in bulk EW and bulk leaf tissue

**Fig. 4.**
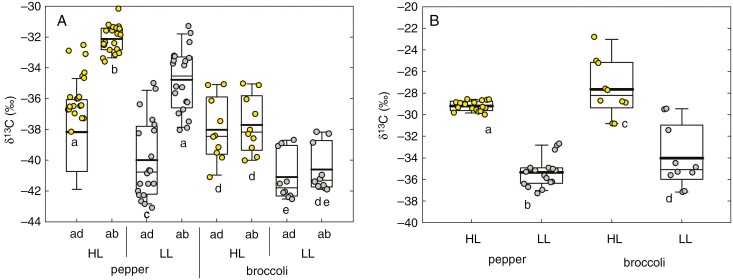
The ^13^C abundance (*δ*^13^C) in pepper and broccoli leaves grown under high (HL, 450 ± 50 μmol m^−2^ s^−1^) and low (LL, 100 ± 20 μmol m^−2^ s^−1^) irradiance. The *δ*^13^C was measured in adaxial (ad) and abaxial (ab) bulk epicuticular wax (A) and in bulk dry mass (B). The lines inside the boxes indicate the median and mean (thin and thick line, respectively); the lower and upper boundaries of boxes and error bars show the 25–75 percentiles and 10–90 percentiles, respectively (pepper, *n* = 20; broccoli, *n* = 10). One-way ANOVA, *P* < 0.05. Separate statistical analyses were performed for pepper and broccoli datasets. Identical letters below the columns indicate the absence of statistically significant differences.

Bulk adaxial EW was depleted in ^13^C (having more negative *δ* values) compared with bulk abaxial EW in both species and light environments (Fig. 4A). The leaf-side differences in *δ*^13^C of EW (*δ*_ab_ − *δ*_ad_) in pepper leaves from both light environments were significant and pronounced, whereas broccoli leaves showed almost identical *δ*^13^C of adaxial and abaxial waxes. The *δ*^13^C values of bulk leaf tissue from HL plants were significantly enriched compared with LL plants in both pepper (*t* = 442, *n* = 17, *P* < 0.001) and broccoli (*t* = 151, *n* = 10, *P* < 0.001) (Fig. 4B). As expected, the wax on both leaf sides and in both species was depleted in ^13^C (more negative) when compared with the respective leaf bulk tissue (Table 1), which indicates discrimination of the heavier carbon isotope during wax synthesis. The δ^13^C difference between bulk leaf tissue and bulk wax was higher in HL than LL plants and higher in broccoli than in pepper (see Table 1).

**Table 1.** Abundance of ^13^C in leaf dry mass (*δ*_DM_) and in bulk epicuticular wax (*δ*_EW_; average of adaxial and abaxial wax) of pepper and broccoli plants grown under high (450 ± 50 μmol m^−2^ s^−1^) and low (100 ± 20 μmol m^−2^ s^−1^) irradiance. The difference between leaf mass and wax (*δ*_DM_ − *δ*_EW_) indicates carbon isotope discrimination during wax synthesis. Data are averages from 10–20 plants grown in four independent experimental runs. A stable isotope ratio mass spectrometer was used for bulk epicuticular wax and dry leaf mass analyses.

Species	Growth irradiance	Leaf dry mass ^13^C content	Leaf epicuticular wax ^13^C content	Leaf mass–wax ^13^C difference
		*δ*_DM_ (‰)	*δ*_EW_ (‰)	*δ*_DM_ − *δ*_EW_ (‰)
*Capsicum annuum*	HL	−28.26	−33.91	5.65
	LL	−33.91	−37.41	3.50
*Brassica oleracea*	HL	−27.63	−37.98	10.35
	LL	−33.95	−40.87	6.92

### Differences in δ^13^C and [CO_2_] between opposite leaf sides; links to amphistomy

**Fig. 5.**
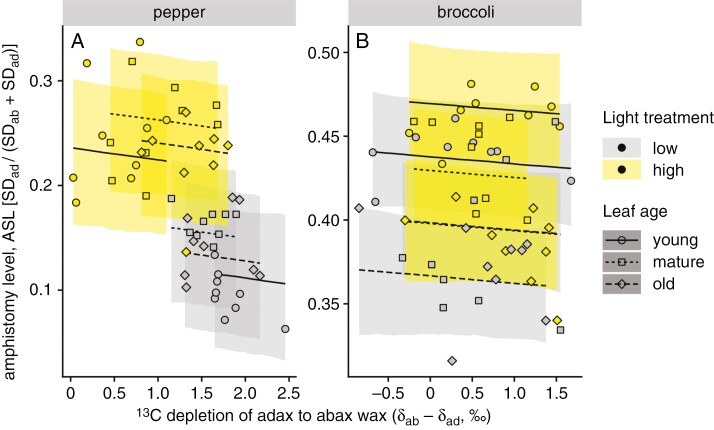
Relationships between amphistomy level (ASL) of pepper (A) and broccoli (B) leaves and the δ^13^C difference between the adaxial and abaxial leaf wax (*δ*_ab_ − *δ*_ad_). Plants were grown at two different light intensities (HL, 450 ± 50 μmol m^−2^ s^−1^, yellow symbols; LL, 100 ± 20 μmol m^−2^ s^−1^, grey symbols); leaves of three insertion levels (ages) were analysed: low (old), middle (mature) and upper (young). Points indicate differences between the leaf sides in *δ* values calculated as the averages weighted by the abundance of individual wax compounds. Lines are linear regressions of ASL on (*δ*_ab_ − *δ*_ad_); ribbons are 95 % confidence bands of the regression.

The approach adopted in experimental runs 1–4, where the leaf age and wax compound factors were not distinguished, failed to address the source of the large variation found in (*δ*_ab_ − *δ*_ad_) (Supplementary Data Fig. S6). In an attempt to reveal the source, in experimental run 5 we introduced additional factors of leaf insertion (age) and the chemical composition of wax. Instead of bulk IRMS, compound-specific isotopic analyses (GC-IRMS) were performed. Consistently with the results in previous experiments, the adaxial EW was depleted in ^13^C against the abaxial EW [(*δ*_ab_ − *δ*_ad_) > 0] in the vast majority of leaves in both species (Fig. 5A, B and Supplementary Data Fig. S7), and ASL values closely matched those of experimental runs 1–4. The effect of light was also conserved: shaded plants had higher (*δ*_ab_ − *δ*_ad_) than lit plants. Growth light, age and their interaction significantly affected the (*δ*_ab_ − *δ*_ad_) values in pepper but not in broccoli (see Table 2 for summary statistics and *P*-values), and this also applies to the [CO_2_] gradient across the leaf (Supplementary Data Fig. S8).

**Table 2.** Statistical significance of the effect of growth light level, leaf insertion level (age) and their interactions on the difference in *δ* (content of ^13^C in epicuticular wax) between opposite leaf sides (*δ*_ab_ − *δ*_ad_), on amphistomy level (ASL), amphiwaxy level (AWL) and stomatal density (SD) on adaxial and abaxial leaf sides. The ASL was expressed as the fraction of adaxial stomata from the total number of stomata on both leaf sides and the AWL as the fraction of epicuticular wax deposited on the adaxial leaf side over total epicuticular wax on both leaf sides. Significant effects (*P* < 0.05) are indicated by bold numbers. The Holm–Sidak test was used in ANOVA.

Species	*δ*_ab_ − *δ*_ad_ (‰)		ASL		AWL		SD_adaxial		SD_abaxial	
	*F*	*P*-value	*F*	*P*-value	*F*	*P*-value	*F*	*P*-value	*F*	*P*-value
*Capsicum annuum*										
Light	56.9	**<0.001**	127.9	**<0.001**	33.5	**<0.001**	15.8	**<0.001**	849.6	**<0.001**
Age	3.5	**0.039**	5.0	**0.010**	2.6	0.085	109.1	**<0.001**	25.8	**<0.001**
Light × age	8.3	**<0.001**	5.3	**0.009**	9.1	**<0.001**	7.4	**<0.001**	14.5	**<0.001**
*Brassica oleracea*										
Light	1.9	0.178	18.2	**<0.001**	0.3	0.571	44.9	**<0.001**	42.4	**<0.001**
Age	1.0	0.367	34.0	**<0.001**	3.6	**0.036**	**71.8**	**<0.001**	**53.0**	**<0.001**
Light × age	0.7	0.506	2.9	0.067	0.1	0.877	1.5	0.237	4.6	**0.015**

As expected and shown in Table 2, ASL increased significantly in HL compared with LL in both species and was affected by age: ASL increased with insertion level from old to young leaves in broccoli under both light regimes (Fig. 5B), but the effect of insertion gradient was not consistent in pepper (Fig. 5A). The ^13^C depletion of adaxial to abaxial wax (*δ*_ab_ − *δ*_ad_) also increased with light in conjunction with ASL. Bayesian path analysis revealed that in pepper, the negative association between ASL and (*δ*_ab_ − *δ*_ad_) is indirect and mediated by shared effects of light treatment (Supplementary Data Table S2). In broccoli, there was less association between ASL and (*δ*_ab_ − *δ*_ad_) because of limited variation in ASL compared with pepper.

### Potential level of amphistomy

**Fig. 6.**
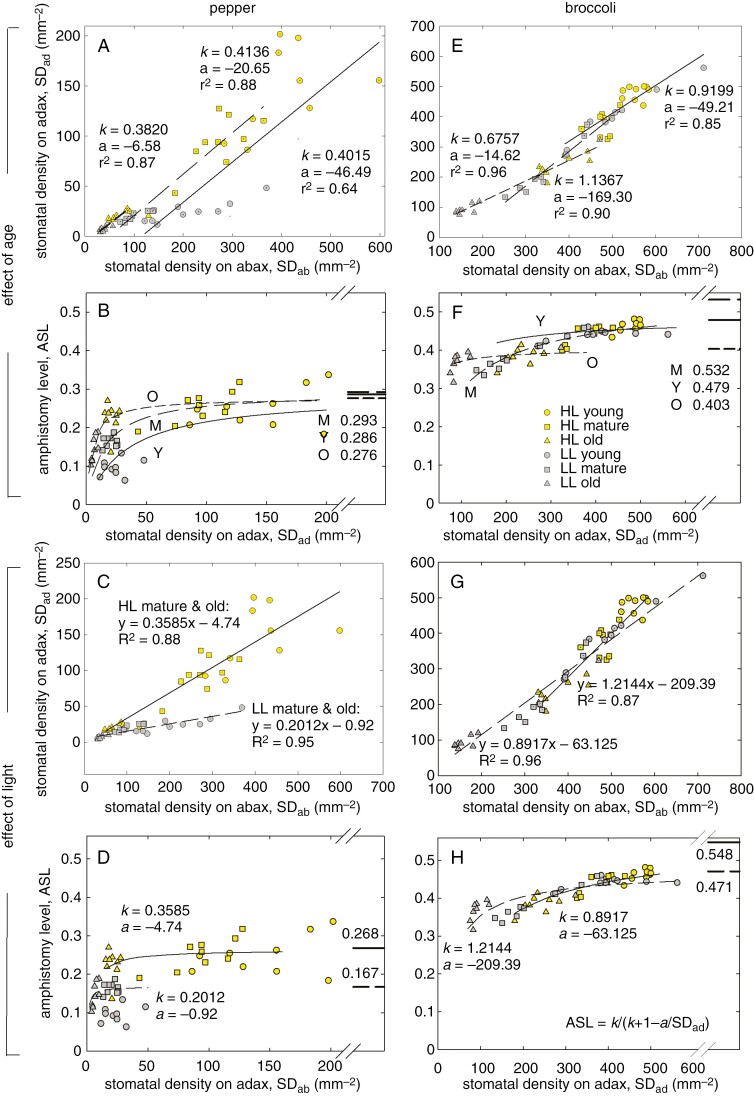
Modelling of potential levels of amphistomy (ASL_pot_) affected by growth light and insertion level (age) of pepper and broccoli leaves. Linear correlation of the upper and lower leaf-side stomatal density (SD_ad_ and SD_ab_, respectively) in young (continuous line), mature (long-dashed line) and old (short-dashed line) leaves of pepper (A) and broccoli (E) across two levels of irradiance [high light (HL) and low light (LL)] is shown. Likewise, the SD_ad_ vs. SD_ab_ linear correlations for plants grown in HL (continuous line) and LL (dashed line) irrespective of leaf age (C, G) are presented together with parameters of regression lines, slopes (*k*) intercepts (*a*) and coefficients of determination (*r*^2^). The parameters *k* and *a* in A and E were used for reconstruction of the light dependence of ASL in young, mature and old leaves of pepper (B) and broccoli (F). The parameters *k* and *a* in C and G allowed prediction of ASL across all the age categories in plants grown in HL and LL conditions (D, H). The thick lines and numbers on the right-hand sides of B, F, D and H indicate asymptotes of ASL, the potential amphistomy level (ASL_pot_). The values of ASL and ASL_pot_ were calculated using the equation shown in H. Stomatal density changes with growth light and insertion level (leaf age) on both leaf sides proportionally (linearly with parameters of slope *k* and intercept *a*). This implies that a maximum of amphistomy (asymptote of ASL) exists, which can be predicted from the slope *k* at SD_ad_→∞. We hypothesize that the ASL asymptote represents the optimal level of amphistomy with respect to the CO_2_ supply and, probably, to the leaf and plant hydraulic limits of water supply or loss.

It is shown above that AS increased with light in both species and with the leaf insertion in broccoli. The increase certainly has a limit, which can change with light and/or leaf age. To resolve this, we modelled ASL using eqn (2) and the linear correlations of SD_ad_ vs. SD_ab_, which accounts for the factor of leaf age across two light regimes in pepper and broccoli (Fig. 6A, E), and the correlation, which follows the factor of light across all three age categories (Fig. 6C, G). The SD_ad_ was correlated linearly with SD_ab_ in all combinations of the categories, with coefficient of determination *R*^2^ ≈ 0.9 (Fig. 6A, C, E, G). The maximum level of AS derived from the correlation lines, ASL_pot_, amounts to 0.27 (HL) and 0.17 (LL) (Fig. 6D); 0.28 (old), 0.29 (mature) and 0.29 (young) in pepper (Fig. 6B); and was higher in broccoli [0.55 (HL) and 0.47 (LL) in Fig. 6H; 0.40 (old), 0.53 (mature) and 0.48 (young) in Fig. 6F].

### Amphiwaxy

**Fig. 7.**
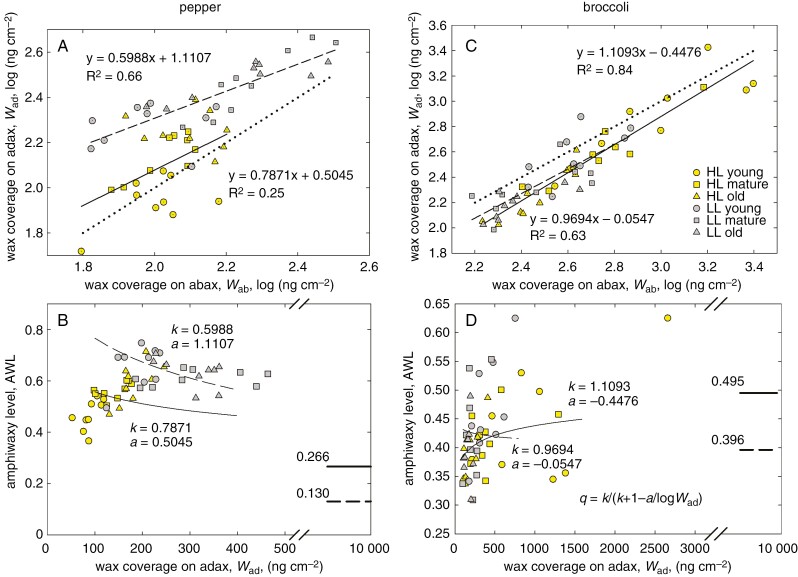
Relationship between epicuticular wax coverage on the opposite leaf sides. Correlations of the wax coverage on the adaxial side (*W*_ad_) with that on the abaxial leaf side (*W*_ab_) in pepper (A) and broccoli (C) and their relationship approximated by linear regression lines are plotted separately for plants grown in high light (HL, continuous line) and low light (LL, dashed line) and include all three age categories. The dotted line represents the 1:1 relationship. Notice that *W* was log-transformed. The line slopes (*k*) and intercepts (*a*) were used in calculation of the parameter *q* using the relationship shown in D and converted to values of amphiwaxy level (AWL) plotted against *W*_ad_ in B and D. The thick lines and respective numbers in B and D show the near-asymptotic potential values of AWL_pot_ obtained at high *W*_ad_ (*W*_ad_ = 10 μg cm^−2^). The depositions of epicuticular wax on adaxial and abaxial sides are related linearly. Slope (*k*) of this coupling called amphiwaxy determines the asymptotic value, maximum (at *k* > 1) or minimum (at *k* < 1) of amphiwaxy, which could potentially be reached at the infinitely high wax deposition on the adaxial side (*W*_ad_→∞). We hypothesize that the adaxial–abaxial patterning of wax coverage (amphiwaxy) and stomata (amphistomy) are interrelated.

**Fig. 8.**
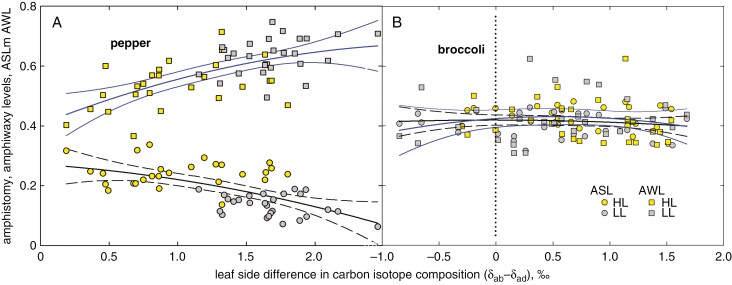
Stomata and epicuticular wax patterning on opposite leaf sides related to the leaf-side difference in ^13^C content of epicuticular wax. Amphistomy level (ASL, circles and black lines) and amhiwaxy level (AWL, squares and blue lines) share common values in broccoli over the whole range of (*δ*_ab_ − *δ*_ad_) values (B), whereas in pepper (A), ASL and AWL converge at the transition from shaded (grey symbols) to sunny (yellow symbols) type of leaves when the (*δ*_ab_ − *δ*_ad_) is approaching zero.

Epicuticular wax deposits on both leaf sides. The amount of EW reported here was determined by using GC-IRMS as the area of peaks summed over all major wax compounds (five alkanes in pepper; three of five most abundant compounds in broccoli). The EW coverage (amount divided by the leaf area) was almost one order of magnitude higher in broccoli than in pepper. It was higher in LL than in HL pepper leaves, whereas the light effect was opposite in broccoli (Supplementary Data Fig. S9). In pepper, significantly more wax deposited on the upper (adaxial) leaf side, whereas the opposite holds for broccoli. Our results have shown that the abaxial–adaxial difference in wax isotopic composition (*δ*_ab_ − *δ*_ad_) varies in parallel to ASL and reaches near-zero values at the plateau of ASL. In search of causal links other than the [CO_2_] to (*δ*_ab_ − *δ*_ad_), we determined the pattern of wax deposition on the opposite leaf sides. The deposition on the adaxial side (*W*_ad_) was linearly proportional to that at the abaxial leaf surface (*W*_ab_), in a similar manner to stomata, albeit with lower *R*^2^ (Fig. 7A, C). The plot shows that EW in pepper was allocated preferably on the adaxial side, but in broccoli on the abaxial leaf side. In analogy to amphistomy, EW patterning can be evaluated as the fraction of EW deposited on the adaxial leaf side over EW on both leaf sides, and we term it the amphiwaxy level (AWL). In broccoli, AWL was similar to ASL over the whole range of (*δ*_ab_ − *δ*_ad_) (Fig. 8B), whereas in pepper, AWL mirrored the relationship of ASL vs. (*δ*_ab_ − *δ*_ad_), decreasing towards the ASL value at near-zero (*δ*_ab_ − *δ*_ad_) (Fig. 8A). The AWL vs. ASL relationship, which also depicts the light and age factors, is shown as the vector plot in Supplementary Data Fig. S10. Effect of light on relation of amphistomy and amphiwaxy level for major epicuticular wax compounds, five alkanes in pepper and three aliphatics in broccoli, is shown in Supplementary Data Fig. S11. The predictions of AWL for very high values of EW deposited on the adaxial side yielded values of AWL_pot_ similar to ASL_pot_ (compare Figs 7B, D and 6D, H).

**Fig. 9.**
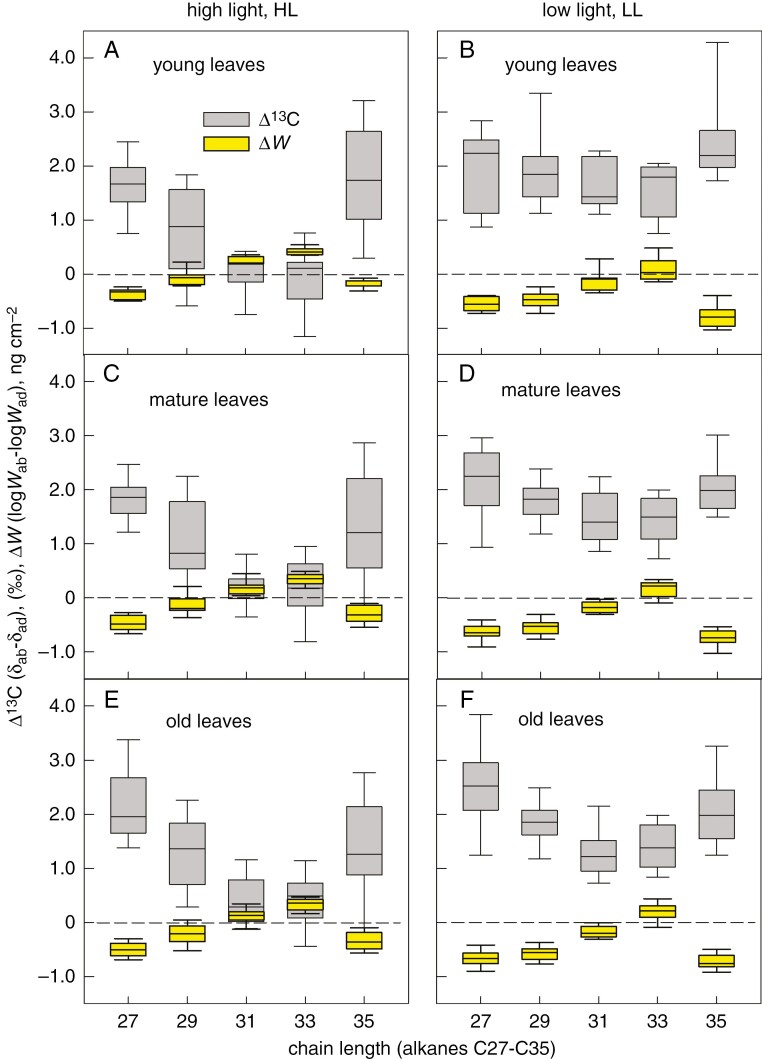
Relationships between the leaf-side-specific differences in deposition of epicuticular wax compounds (Δ*W*) of pepper and the leaf-side-specific differences in their ^13^C content (Δ^13^C). Five odd-chain alkanes (C27, C29, C31, C33 and C35), extracted by *n*-hexane from epicuticular wax of adaxial and abaxial pepper leaves, were investigated for their amount (*W*, determined by gas chromatography) and carbon isotope ratio (*δ*). Plants were grown at high light (A, C, E) or low light (B, D, F) and leaves of three age categories - young (A, B), mature (C, D) and old (E, F) - were investigated. The abaxial and adaxial *W* is expressed as log*W*. The line inside the boxes indicates the median; the lower and upper boundaries of boxes and error bars show the 25–75 and 10–90 percentiles, respectively. Each box was calculated from nine measurements on three plants.

The differences in EW amount, Δ*W* = *W*_ab_ − *W*_ad_, and in isotopic signature, Δ*δ* = (*δ*_ab_ − *δ*_ad_), between the leaf sides refined to the individual pepper alkanes confirmed the general trend: Δ*W* and Δ*δ* behaved in a complementary manner in all age categories and both light treatments (Fig. 9A–F). Compounds approaching zero in Δ*δ* deposited equally on both leaf sides (Δ*W* = 0).

## DISCUSSION

### Amphistomy, a trait indicating better access of CO_2_ into the leaf

The adaptive significance of amphistomy is not fully understood, but it is likely to provide the benefits of opening up an additional pathway for CO_2_ through the upper leaf side and an anticipated increase of [CO_2_] inside the light-exposed part of the mesophyll (Parkhurst, 1978; Mott *et al.*, 1982; Drake *et al.*, 2019; Triplett *et al.*, 2024). The results presented here indicate that this benefit of AS can be detected using the isotopic polarity of leaf cuticular wax, (*δ*_ab_ − *δ*_ad_), as a proxy for the [CO_2_] gradient across the leaf. We have shown that the gradient that forms between the lower stomatous and the upper astomatous side in HS leaves and probably imprints in (*δ*_ab_ − *δ*_ad_) develops in the opposite direction in herbaceous AS plants grown in shade and disappears in sunlit AS species. The gradients in shade-grown leaves, which differed markedly between the HS and AS plant species, varied with the ASL. This is in accordance with our hypothesis that the development of stomata is co-determined by the [CO_2_] inside the leaf (Santrucek *et al.*, 2014). We assume that imprinting of the [CO_2_] gradient is based on the unique ability of EW to record [CO_2_] in epidermal plastids and adjacent chloroplasts in its carbon isotope composition. This phenomenon was identified only recently (Santrucek *et al.*, 2019); therefore, certain assumptions underlying its application deserve critical examination and testing.

#### Wax compounds archive information about the adjacent intracellular [CO_2_]

We assume that, despite the post-photosynthetic discrimination during lipid biosynthesis, the individual wax compounds and/or bulk wax preserve the information on [CO_2_] in plastids. At least two lines of evidence indicate that this assumption is plausible.

**Fig. 10.**
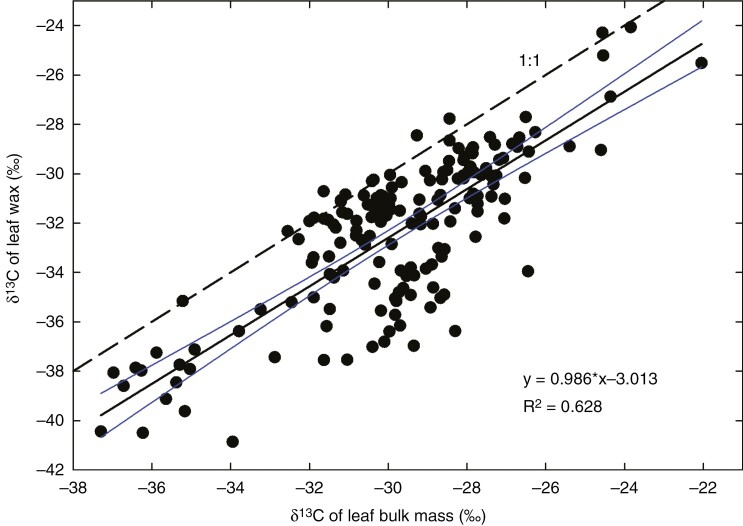
Relationship between carbon isotope compositions of leaf cuticular wax and bulk leaf dry mass. Data were collected from 178 stable isotope ratio mass spectrometer analyses of bulk leaf dry mass and wax of 49 hypostomatous and four amphistomatous C_3_ species in our laboratory between 2004 and 2021. Each point represents the mean of *δ*^13^C for the adaxial and abaxial wax, plotted vs. *δ*^13^C of the leaf dry mass. The dashed line shows the 1:1 relationship (i.e. *δ*^13^C being identical in wax and leaf dry mass). The continuous line represents the regression (see inset equation) together with its 95 % confidence interval.

First, *δ*^13^C of bulk wax and its main constituents parallels *δ*^13^C of leaf dry mass in a broad set of C_3_ species grown in a variety of environmental conditions (Fig. 10). The main source of variability in *δ*^13^C of plant assimilates (mainly cellulose) is the variation in leaf internal [CO_2_], *c*_i_ (Farquhar *et al.*, 1982; Cernusak *et al.*, 2013). Therefore, the co-variation of *δ*^13^C in wax and leaf biomass indicates that the information on *c*_i_ is preserved in cuticular wax despite the shift caused by post-photosynthetic discrimination during wax synthesis (−3.0 ‰ in our set of data).

Second, it would appear to be an unlikely coincidence that wax on the adaxial epidermis of HS species is almost always enriched in ^13^C compared with the abaxial wax (Figs 1 and 10; for other HS species, cf. Santrucek *et al.*, 2019). Such isotopic polarity of cuticular wax corresponds to the [CO_2_] gradient in HS leaves postulated by gas exchange theory (diffusion of CO_2_ along the gradient of [CO_2_] through mesophyll). It is even less likely that the HS isotopic polarity would turn into its inverse in AS leaves without being related to leaf-side-specific stomatal distribution. It must be admitted that we encountered a few exceptions to this rule, whereby wax of an HS species showed features of AS, i.e. it was depleted in ^13^C on the upper vs. lower leaf side. For more information, see the Supplementary Data under ‘Cases contrary to the rule’ and ‘Alternative sources of leaf wax isotopic polarity beyond CO_2_ concentration gradient’, including Supplementary Data Fig. S12.

#### Wax compounds and their precursors are not amalgamated in the leaf or plant body

In an individual cell, fatty acids are exchanged at a massive rate via a transport mechanism between plastids and the endoplasmic reticulum. The exchange occurs in both directions. However, each plant cell is largely autonomous in lipid biosynthesis; glycerolipids and fatty acids are not transported between cells at an extensive scale (Somerville *et al.*, 2000). Epidermal cells are a remarkable exception to this rule. Very-long-chain wax compounds and C_16_ or C_18_ oxygenated fatty acids as monomers of cutin are transferred from the endoplasmic reticulum to outside the cell and deposited in and on the cuticle; however, details of the transport mechanism are not fully understood (Kunst and Samuels, 2009). The inherent barriers for cell-to-cell transport of lipids, with the single exception of epidermal cells, make the assumption in subtitle realistic, at least for mature fully autotrophic leaves.

#### Wax deposits in cuticle continually during the whole leaf lifespan

It is important to consider when in leaf ontogeny wax and cuticle components are synthesized and deposited. If it happens before the leaf acquires full autotrophy, the cuticle and wax might be synthesized from mixed sugars imported from mature plant leaves and thus be lacking any leaf-side specificity. In contrast, if wax is synthesized and re-deposited continually during the whole leaf lifespan, it might record changes in the leaf internal environment, namely [CO_2_]. The timing of deposition is likely to differ for various cuticle constituents. Cutin, polysaccharides and other compounds forming cuticular matrix are probably deposited early during the leaf development with limited, if any, renewal (Kubásek *et al.*, 2023). However, wax embedded in the matrix (intracuticular) and especially that deposited on the surface (epicuticular) is restored during the entire leaf lifespan, even after the leaf reaches its full size (Kubásek *et al.*, 2023). Hauke and Schreiber (1998) showed that leaves of *Hedera helix* reached their maximum area 30–40 days after bud burst, whereas the mass of the cuticle increased to a plateau reached between 60 and 90 days after bud burst (i.e. the thickness of cuticle increased). Pulse labelling with stable hydrogen or carbon isotopes revealed that wax seems to be highly dynamic in terms of loss (by abrasion and washing off) and *de novo* synthesis. By using deuterium labelling, Gao *et al.* (2012) estimated the recycling time of grass leaf waxes (*Phleum pratense*) to be of the order of weeks. The turnover time of wax compounds varied according to their chain length from 5–16 days (C22–C26) to 71–128 days (C27–C31) and was even shorter for C16–C18 precursors of cutin (2–3 days). Jetter and Schaffer (2001) published data showing continuous *de novo* synthesis of EW during 60 days of *Prunus laurocerasus* leaf development. In light of this information, we believe that cuticle and especially EW renew dynamically and can integrate information on intracellular conditions with a resolution of days to months for the whole leaf lifespan.

### Coordination of stomata development and wax deposition on adaxial and abaxial sides

Amphistomatous species usually increase the proportion of adaxial stomata in response to stronger light. This corresponds to the tendency of stomata to respond in parallel for the capacity of mesophyll to assimilate CO_2_ (Mott and Peak, 2018). In coordination with newly developing stomata, an airspace is created between mesophyll cells (Lundgren *et al.*, 2019). Concomitantly, leaf anatomy and/or morphology changes towards thicker, more compact and usually smaller blades, with denser venation (Mott and Michaelson, 1991; Terashima *et al.*, 2006; Drake *et al.*, 2019). Undoubtedly, the development of thicker sun-exposed leaves, with a more complex anatomy (Fig. 2), more stomata per unit projected leaf area (Fig. 3) and usually with thicker cuticle, induces additional costs compared with shaded leaves (Drake *et al.*, 2019). It raises the questions: what is the maximal level of amphistomy (ASL_pot_), and how are the benefits and costs balanced? Interestingly, the SD of adaxial and abaxial sides responds to light or age in a coordinated way, as indicated by their linear correlations (Fig. 6A, C, E, G). The ASL derived from the linear response approaches maximum values, ASL_pot_, specific for species and growth conditions (Fig. 6B, D, F, H). Remarkably, the opposite leaf sides also coordinate EW deposition and reach amphiwaxy maxima, AWL_pot_, in a similar manner to ASL_pot_ (Fig. 7A–D). Moreover, ASL and AWL converge to a similar value at (*δ*_ab_ − *δ*_ad_) approaching zero in pepper (Fig. 8A). What is the physiological role (if any) of the stomata and wax parallel response?

Although we found only an indirect light-mediated relationship between ASL and (*δ*_ab_ − *δ*_ad_), it is tempting to speculate that ASL and AWL reach a common asymptotic value when both sides of the leaf experience identical *δ* and, thus, equal leaf internal [CO_2_]. In such a state, termed ‘optimal ASL’ here, the increased proportion of adaxial stomata would match the increased mesophyll, namely palisade parenchyma, demand for CO_2_ at incremented light. For example, the lines for the four AS species presented in Fig. 1 indicate by their intercepts at (*δ*_ab_ − *δ*_ad_) = 0 that ASL is 0.23, 0.45, 0.47 and 0.58 for pepper, broccoli, broadleaf plantain and bunya pine, respectively. This would mean that CO_2_ access and assimilation fluxes on both sides were balanced when about 23, 45, 47 and 58 %, respectively, of all stomata per unit projected leaf area were allocated to the upper epidermis. Detailed experiments with pepper and broccoli confirmed these intercept ASL values. We are aware that the above concept of asymptotic maximal value of amphistomy might be oversimplified. The relationship between environment (light) and ASL is not necessarily linear (Muir, 2019) and comprises potentially different responsivities of stomata on opposite leaf sides (Richardson *et al.*, 2017; Wall *et al.*, 2022). Additional costs of AS (e.g. for water uptake and transport) and diminishing increments in photosynthetic benefit with increasing light might underlie the lack of additional adaxial stomata developed.

**Fig. 11.**
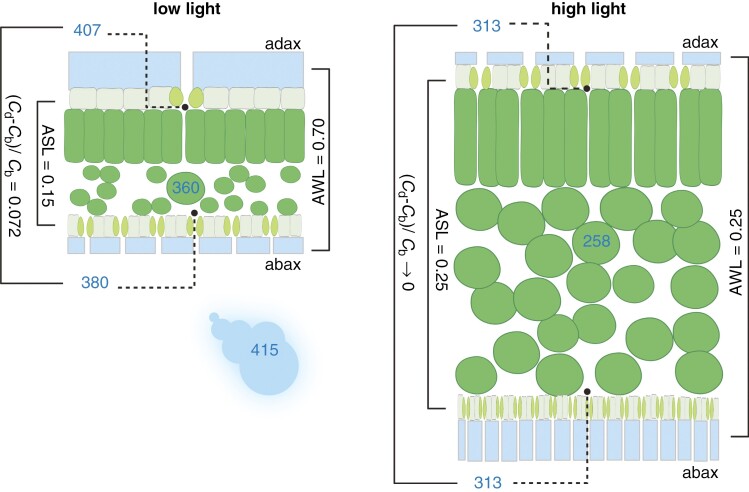
Diagram summarizing typical values in stomatal density, epicuticular wax deposition and intercellular [CO_2_] on opposite sides of pepper leaves at two growth irradiances. Palisade and spongy mesophyll (dark green), epidermes (grey) with stomata guard cells (light green) and epicuticular wax (EW, blue) are shown. Stomatal density and the amount of EW on the adaxial and abaxial surfaces are indicated in relative proportions within each leaf and between light treatments, as is the thickness of the leaf grown in low-light (LL, left) and high-light (HL, right) conditions. The thickness of wax and epidermis are not in proportion to the thickness of mesophyll. Fractions of stomata and EW deposited on the upper (adaxial) and lower (abaxial) sides, amphistomy and amphiwaxy levels (ASL and AWL, respectively) correspond to the values found here and indicate that both stomata and wax patterns are identical in HL leaves. Blue numbers indicate [CO_2_] (in micromoles per mole) as follows: (1) in ambient atmosphere (415) common for both light treatments; (2) inside the leaf, calculated from *δ*^13^C of the leaf dry mass assuming infinite mesophyll conductance for CO_2_ (360 and 258 in LL and HL leaves, respectively); and (3) in substomatal adaxial and abaxial intercellular space. The last of these is an example calculated on the assumption that ~60 % of the difference in [CO_2_] between the leaf and the atmosphere is accounted for by the stomatal resistance and 40 % by the mesophyll resistance, while the difference between adaxial and abaxial concentrations corresponds to that measured from the leaf-side difference in isotopic composition of cuticular wax. The figure was created with BioRender.com.

Although wax coverage (load), its correlation with the leaf transpiration rate or dependence on environmental factors, especially drought, has often been the matter of interest (Riederer and Schreiber, 2001; Grunhofer *et al.*, 2022), it was seldom investigated separately for both leaf sides (Premachandra *et al.*, 1993; Wen *et al.*, 2006). The significance and mechanism of the coordinated response in wax deposition and stomata development has only recently begun to be studied (Gray *et al.*, 2000; Aharoni *et al.*, 2004; Khoudi, 2023) and revealed on the genome level (Liu *et al.*, 2022). In shade-acclimated pepper, wax deposited relatively more on the adaxial side and was depleted in ^13^C compared with wax on the abaxial side. With increasing density of stomata on the adaxial side under HL, and thus with rising ASL, wax starts to deposit relatively more on the abaxial side, and the isotopic difference between leaf sides diminishes (Fig. 8A). The light effect on bulk EW partitioning was consistent with the response of individual compounds (Supplementary Data Fig. S11) and, moreover, the link between stomata pattern and leaf-side specificity of the wax load was general for both light (Supplementary Data Fig. S13B) and leaf insertion level (Supplementary Data Fig. S13A). A schematic diagram summarizing the changes in stomata distribution, wax partitioning and [CO_2_] drawdowns at the opposite leaf sides and at the transition from shaded to amply lit pepper plants is shown in Fig. 11.

We can only speculate about the physiological benefits of the changes in EW patterning occurring concomitantly with variation of ASL. Functional properties of EW and CO_2_ access into the leaf are likely to underlie the reasons. It has been reported that the EW does not form the transpiration-limiting barrier; the function, which is usually attributed to intracuticular wax (Zeisler and Schreiber, 2016). However, these results were obtained with astomataous adaxial leaf cuticles and adaxial EW. Zhang *et al.* (2020) confirmed the inactivity of adaxial EW in reducing cuticular transpiration in *Camellia sinensis* but reported that the abaxial EW does constitute the transpiration barrier. If EW performs differently on the opposite leaf sides, this might explain why EW deposits more on the abaxial side of HL pepper with an increasing number and changing distribution of stomata. The cuticle on the stomatous leaf surface, or on the surface with elevated stomatal density, is more prone to water loss than the astomatous one (Santrucek *et al.*, 2004; Karbulkova *et al.*, 2008), and additional EW could contribute to its sealing.

## Conclusion

In conclusion, the most significant phenomenon described for the first time here is that hypostomy differs from amphistomy in the carbon isotope dorsiventral polarity of EW, (*δ*_ab_ − *δ*_ad_). This study provides indirect, *δ*^13^C-based evidence that AS is connected with the benefit of enhanced CO_2_ flux through the upper (adaxial) leaf epidermis. Pepper, which responds to increased light with pronounced enhancement of ASL, provides a good example of the relationships between stomata and EW partitioning and concomitant changes in [CO_2_] drawdowns from ambient atmosphere into adaxial and abaxial leaf mesophyll (Fig. 11). The drawdown of [CO_2_] was smaller at the adaxial than abaxial leaf side in three herbaceous species grown in LL conditions. With increased ASL and leaf thickness in plants grown in HL conditions, the values of CO_2_ drawdowns on both leaf sides tended to become the same (*δ*_ab_ − *δ*_ad_ = 0) and, presumably, indicate the optimal level of AS. Given that the leaf internal [CO_2_] decreased in the HL-grown plants, the [CO_2_] drawdown inevitably increased, which, together with higher stomatal density on both leaf sides, contributed to enhanced CO_2_ assimilation rate. The adaptive significance of such a state, accompanied by adjustments of ASL, EW partitioning, leaf anatomy and, probably, also by adjustment of mesophyll conductance for CO_2_, leaf hydraulic conductance, photosynthetic capacity and leaf optical properties, might lie in an improved balance of the benefits and costs of leaf function. The leaf-side-specific patterning of EW, amphiwaxy level (AWL), is the newly observed leaf trait linked to the state of ASL. Interestingly, ASL and AWL approached similar values in pepper and broccoli grown under HL and LL. The adaptive significance of EW patterning could stem from the leaf-side-specific functional properties of EW.

## SUPPLEMENTARY DATA

Supplementary data are available at *Annals of Botany* online and consist of the following.

Text parts: cases contrary to the rule, alternative sources of leaf wax isotopic polarity beyond [CO_2_] gradient. Table S1: relative abundance and *δ*^13^C of wax components isolated from the adaxial and abaxial sides of leaves of five hypostomatous (HS) and four amphistomatous (AS) species. Table S2: summary of parameter estimates from Bayesian path analytical model for broccoli (A) and pepper (B). Figure S1: twigs with amphistomatous needles of bunya pine (*Araucaria bidwillii*) and leaves of hypostomatous karri tree (*Eucalyptus diversicolor*). Figure S2: spectral composition of incident light used during growth of broccoli and pepper. Figure S3: examples of pepper (*Capsicum annuum* L.) and broccoli (*Brassica oleracea* L., var. *italica*) plants. Figure S4: abundance of major compounds of epicuticular wax (EW) isolated from leaf surfaces of pepper (A) and broccoli (B). Figure S5: stomatal density and amphistomy level of pepper and broccoli leaves. Figure S6: the differences in isotopic composition between abaxial and adaxial epicuticular waxes (*δ*_ab_ − *δ*_ad_) (A) and in leaf internal [CO_2_] across the leaf [(*c*_ad_ − *c*_ab_)/c_ab_] (B) plotted against amphistomy level (ASL). Figure S7: carbon isotope ratio (*δ*^13^C) of adaxial epicuticular wax (EW) as related to *δ*^13^C of abaxial EW in pepper and broccoli. Figure S8: relationships between amphistomy level (ASL) of pepper (A) and broccoli (B) leaves and the relative drawdown of [CO_2_] across the leaf [(*c*_ad_ − *c*_ab_)/*c*_ab_] calculated using eqn (1). Figure S9: epicuticular wax coverage on adaxial and abaxial leaf sides of pepper and broccoli grown at two different irradiances. Figure S10: the effect of light on amphistomy level (ASL) and amphiwaxy level (AWL) in pepper and broccoli leaves at three ontogeny stages. Figure S11: effect of light on relationship of amphistomy and amphiwaxy levels for major epicuticular wax compounds, five alkanes in pepper and three aliphatics in broccoli. Figure S12: relationship between the depletion of epicuticular wax in ^13^C against leaf dry mass (*δ*_DM_ − *δ*_wax_) and the wax coverage. Figure S13: relationship between stomatal density and partitioning of epicuticular wax between the opposite sides of pepper leaf.

mcae082_suppl_Supplementary_Material
