# Supplementary material for: Amphistomy: stomata patterning inferred from 13C content and leaf-side-specific deposition of epicuticular wax
Source: Ann Bot. 2024 Jun 5;134(3):437–54. doi: 10.1093/aob/mcae082 (PMC11341673; doi:10.1093/aob/mcae082)
Supplement: mcae082_suppl_Supplementary_Material [file mcae082_suppl_supplementary_material.docx]

**Supplementary Information**

***Cases contrary to the rule***

The most significant phenomenon described for the first time here is that hypostomy (HS) differs from amphistomy (AS) in the carbon isotope dorsiventral polarity of epicuticular wax, δ_ab_-δ_ad_. While this observation applies to the herbaceous AS plants investigated here, the hard broad-needle AS conifer, *Araucaria* *bidwillii* (bunya pine), behaved like a HS species when grown in deep shade (Fig. 1). What is behind this “anomaly”? Leaves growing in the deep shade of the understorey of tropical forests obtain a significant amount of carbon by assimilation during transient sunflecks, i.e. short periods of bright sunshine (Way and Pearcy, 2012). Fast responding stomata are advantageous for this type of carbon assimilation. As shown by Haworth et al. (2018) in a survey of 31 species, the more dynamic type of stomatal response occurs in AS species more often than in HS ones. Therefore, allocation of stomata to the upper epidermis, although not beneficial through most of the day when wax is synthetized, could bring a functional advantage during sunflecks, especially in the humid air conditions of a rain forest. Thus, it appears that the bunya pine in deep shade, although AS, behaves like a HS for most of the day and only during sunflecks does the CO_2_ concentration gradient within its leaf change. However, wax synthesis is continuous and not limited to the periods of sunflecks. Another and so far single anomaly, which we systematically observed in the leaf-side specific ^13^C signature of wax, was authograph tree (*Clusia rosea*). *Clusia* is hypostomatous but its wax isotopic polarity contradicted to the expected enrichment of adaxial wax in ^13^C (data not shown). Perhaps very low photosynthesis rate relatively to respiration in this species and re-assimilation or respiratory CO_2_ could cause the unexpected gradient.

***Alternative sources of leaf wax isotopic polarity beyond CO_2_ concentration gradient***

The path analysis models applied to our results shows no direct association between ASL and gradient of [CO_2_] across the leaf as inferred from the (δ_ab_-δ_ad_) difference. Instead, with paprika, it shows that light treatment induces parallel changes in both ASL and (δ_ab_-δ_ad_). However, within a light treatment there was no effect of ASL on (δ_ab_-δ_ad_) and [CO_2_] gradient. The absence of direct relationship between ASL and (δ_ab_-δ_ad_) at a given light environment can be explained by inherent tendency of individual plant to keep leaf internal [CO_2_] invariable (Wong et al., 1985). However, there is still some probability that the differences in wax isotope composition on opposite sides of the leaf are due to some unknown factor other than CO_2_ concentration. Among environmental factors, differences in temperature or irradiance of the adaxial and abaxial sides of the leaf should be considered. To the best of our knowledge, there are no data in the literature on the carbon isotopic composition of cuticular wax as a function of irradiance or temperature that would simultaneously exclude the effect of CO_2_ concentration in chloroplasts. If site-specific temperature or irradiance were to play a role, it would be difficult to explain why amphi- and hypostomatic leaves have fundamentally different isotopic polarity.

Recently it was shown that the lipid ^13^C depletion against primary sugars or bulk leaf mass can decrease with increasing total wax content in several C_3_ and C_4_ plant species (Zhou et al., 2015). It is known for a long time, that changes in the rate of carbon flux through branching points in lipid metabolism may shape the carbon isotope composition of lipids (Park and Epstein, 1961, DeNiro and Epstein, 1977). Thus, we have checked whether the ^13^C depletion of EW against the leaf dry mass correlates with wax coverage. The coefficients of determination R^2^ were very low in both pepper and broccoli (Fig. S12) indicating that the ^13^C depletion of EW was not driven by the leaf-side specific wax deposition.

**Table S1**

Relative abundance and δ^13^C of wax components isolated from the adaxial and abaxial sides of leaves of five hypostomatous (HS) and four amphistomatous (AS) species. One HS and the four AS species were grown in or collected from high-light and low-light conditions. The wax compounds, identified by their Retention indices (RI), are very-long-chain aliphatic fractions, which are dissolvable in chloroform. The number of carbon atoms in the chain of alkanes can be approximated from the first two digits of the RI number. The bulk of the mass of wax is composed by compounds with odd-numbered carbon chains, mainly alkanes (e.g. RI 2900, 3100, 3300). *Sum* is the fraction of the major compounds (in %) in the total amount of GC-detected compounds; **w.a.** stands for the mean δ^13^C weighted by the abundance of the particular compound.

|  | Sunny leaves | |  |  |  | Shaded leaves | |  |  |  |
| --- | --- | --- | --- | --- | --- | --- | --- | --- | --- | --- |
| Compound | abax |  | adax |  | abax-adax | abax |  | adax |  | abax-adax |
| RI | abundance % | ẟ^13^C_wax | abundance % | ẟ^13^C_wax | ẟ^13^C_AB-AD_ | abundance % | ẟ^13^C_wax | abundance % | ẟ^13^C_wax | ẟ^13^C_AB-AD_ |
| ***Hypostomatous species*** | | |  |  |  |  |  |  |  |  |
| ***Euonymus japonica cv. Katya*** | | | |  |  |  |  |  |  |  |
| 3100 | 32.7 | -37.91 | 3.1 | -37.09 | -0.81 |  |  |  |  |  |
| 3300 | 19.9 | -38.96 | 3.5 | -37.66 | -1.31 |  |  |  |  |  |
| 3450 | 11.5 | -38.10 | 5.8 | -36.84 | -1.26 |  |  |  |  |  |
| 3543 | 9.8 | -35.69 | 50.7 | -34.28 | -1.40 |  |  |  |  |  |
| 3643 | 16.4 | -39.79 | 11.7 | -39.96 | 0.17 |  |  |  |  |  |
| *sum***, w.a.** | *90.3* | **-38.27** | *75.0* | **-35.65** | **-2.62** |  |  |  |  |  |
| ***Schefflera dendricola*** | | |  |  |  |  |  |  |  |  |
| 2900 | 10.40 | -34.34 | 14.12 | -31.85 | -2.49 |  |  |  |  |  |
| 3100 | 12.20 | -39.81 | 7.92 | -36.68 | -3.13 |  |  |  |  |  |
| 3248 | 8.81 | -32.50 | 16.80 | -31.85 | -0.65 |  |  |  |  |  |
| 3451 | 12.16 | -36.70 | 19.55 | -34.88 | -1.82 |  |  |  |  |  |
| 3644 | 3.09 | -38.73 | 2.96 | -36.86 | -1.87 |  |  |  |  |  |
| *sum***, w.a.** | *46.7* | **-36.33** | *61.3* | **-33.68** | **-2.65** |  |  |  |  |  |
| ***Zamioculcas zamiifolia*** | | |  |  |  |  |  |  |  |  |
| 2900 | 9.90 | -42.69 | 6.26 | -39.10 | -3.59 |  |  |  |  |  |
| 3100 | 71.83 | -42.82 | 32.21 | -42.46 | -0.36 |  |  |  |  |  |
| 3245 | 1.57 | -38.41 | 8.10 | -37.66 | -0.75 |  |  |  |  |  |
| 3455 | 4.61 | -39.15 | 28.56 | -39.98 | 0.83 |  |  |  |  |  |
| *sum***, w.a.** | *87.9* | **-42.53** | *75.1* | **-40.72** | **-1.81** |  |  |  |  |  |
| ***Eucalyptus diversicolor*** | | |  |  |  |  |  |  |  |  |
| 1953 | 1.38 | -33.43 | 3.06 | -31.52 | -1.91 | 1.09 | -33.65 | 4.20 | -32.05 | -1.60 |
| 2500 | 1.36 | -34.12 | 5.86 | -32.86 | -1.26 | 0.55 | -34.60 | 3.23 | -34.10 | -0.50 |
| 2633 | 7.83 | -33.25 | 4.14 | -32.68 | -0.57 | 9.15 | -35.83 | 4.76 | -34.65 | -1.18 |
| 2700 | 1.21 | -34.09 | 8.33 | -32.34 | -1.76 | 0.54 | -34.42 | 3.43 | -33.75 | -0.67 |
| 2836 | 39.66 | -33.90 | 16.31 | -34.36 | 0.46 | 49.37 | -35.76 | 22.13 | -34.50 | -1.26 |
| 3000 | 7.07 | -34.39 | 4.37 | -32.70 | -1.69 | 10.96 | -34.95 | 3.87 | -34.44 | -0.50 |
| 3041 | 14.70 | -34.33 | 9.48 | -33.94 | -0.39 | 17.24 | -35.71 | 5.22 | -34.71 | -1.00 |
| *sum***, w.a.** | *73.2* | **-33.97** | *51.5* | **-33.34** | **-0.62** | *88.9* | **-35.61** | *46.8* | **-34.23** | **-1.38** |
| ***Prunus laurocerasus*** | | |  |  |  |  |  |  |  |  |
| 2700 | 5.35 | -37.55 | 4.06 | -35.21 | -2.34 |  |  |  |  |  |
| 2800 | 2.20 | -37.50 | 2.20 | -34.36 | -3.15 |  |  |  |  |  |
| 2900 | 64.25 | -36.67 | 57.53 | -35.22 | -1.44 |  |  |  |  |  |
| 3000 | 2.60 | -36.61 | 2.58 | -34.28 | -2.34 |  |  |  |  |  |
| 3100 | 24.81 | -36.38 | 31.94 | -35.16 | -1.21 |  |  |  |  |  |
| *sum***, w.a.** | *93.9* | **-36.61** | *94.3* | **-35.16** | **-1.45** |  |  |  |  |  |
| ***Amphistomatous species*** | | |  |  |  |  |  |  |  |  |
| ***Plantago sp.*** | |  |  |  |  |  |  |  |  |  |
| 2700 | 4.55 | -30.08 | 5.24 | -34.82 | 4.73 | 4.65 | -33.49 | 5.49 | -34.91 | 1.42 |
| 2900 | 15.35 | -35.44 | 21.88 | -36.07 | 0.63 | 15.60 | -36.31 | 17.61 | -36.66 | 0.35 |
| 3100 | 41.37 | -37.42 | 51.47 | -37.97 | 0.55 | 37.21 | -37.51 | 44.35 | -38.21 | 0.71 |
| 3300 | 21.63 | -37.94 | 12.71 | -36.79 | -1.15 | 22.69 | -37.43 | 18.56 | -37.29 | -0.14 |
| 3500 | 9.73 | -37.77 | 3.33 | -35.64 | -2.13 | 13.20 | -37.83 | 7.89 | -38.79 | 0.96 |
| *sum***, w.a.** | *92.6* | **-36.89** | *94.6* | **-37.12** | **0.23** | *93.4* | **-37.13** | *93.9* | **-37.59** | **0.46** |
| ***Araucaria bidwillii*** | |  |  |  |  |  |  |  |  |  |
| 3100 | 30.16 | -32.78 | 35.91 | -32.51 | -0.27 | 24.77 | -37.92 | 29.80 | -36.73 | -1.19 |
| 3300 | 32.69 | -32.21 | 29.95 | -32.24 | 0.03 | 36.51 | -38.41 | 32.65 | -36.49 | -1.92 |
| 3452 | 9.22 | -30.35 | 8.85 | -30.75 | 0.39 | 13.12 | -36.09 | 12.56 | -34.86 | -1.23 |
| 3500 | 14.26 | -31.56 | 11.40 | -31.10 | -0.46 | 13.63 | -37.37 | 11.35 | -36.36 | -1.01 |
| *sum***, w.a.** | *86.3* | **-32.10** | *86.1* | **-32.05** | **-0.06** | *88.0* | **-37.76** | *86.4* | **-36.32** | **-1.45** |
| ***Brassica oleracea*** | |  |  |  |  |  |  |  |  |  |
| 2700 | 0.54 | -35.46 | 0.66 | -36.09 | 0.63 | 0.65 | -38.62 | 0.79 | -39.87 | 1.25 |
| 2800 | 2.83 | -36.31 | 3.35 | -35.62 | -0.69 | 3.21 | -39.79 | 6.07 | -39.81 | 0.02 |
| 2900 | 43.15 | -36.66 | 43.97 | -36.59 | -0.07 | 42.59 | -40.26 | 41.17 | -40.58 | 0.32 |
| 3087 | 37.54 | -36.66 | 33.95 | -36.48 | -0.17 | 32.27 | -40.62 | 28.32 | -40.75 | 0.13 |
| 3044 | 7.49 | -35.65 | 7.38 | -35.17 | -0.48 | 9.84 | -39.79 | 12.12 | -39.94 | 0.16 |
| 3100 | 3.06 | -39.10 | 2.57 | -37.72 | -1.38 | 7.13 | -40.18 | 5.25 | -40.52 | 0.34 |
| *sum***, w.a.** | *94.6* | **-36.64** | *91.9* | **-36.43** | **-0.21** | *95.7* | **-40.30** | *93.7* | **-40.49** | **0.19** |
| ***Capsicum annum*** | |  |  |  |  |  |  |  |  |  |
| 2700 | 2.04 | -35.53 | 5.37 | -36.66 | 1.14 | 2.59 | -35.65 | 10.33 | -37.40 | 1.75 |
| 2900 | 3.05 | -36.64 | 4.28 | -36.60 | -0.05 | 3.57 | -36.07 | 7.72 | -36.90 | 0.82 |
| 3100 | 15.27 | -36.97 | 16.09 | -36.94 | -0.03 | 14.15 | -35.09 | 24.60 | -36.13 | 1.04 |
| 3200 | 6.12 | -37.79 | 5.37 | -38.81 | 1.02 | 3.11 | -35.64 | 0.80 | -36.27 | 0.63 |
| 3300 | 32.83 | -37.81 | 20.00 | -37.25 | -0.55 | 35.42 | -35.08 | 12.61 | -36.15 | 1.07 |
| 3500 | 2.73 | -38.48 | 7.63 | -38.11 | -0.37 | 3.82 | -35.90 | 9.55 | -36.27 | 0.37 |
| *sum***, w.a.** | *62.0* | **-37.49** | *58.7* | **-37.32** | **-0.18** | *62.7* | **-35.24** | *65.6* | **-36.45** | **1.20** |

**Table S2**

Summary of parameter estimates from Bayesian path analytic model for (A) broccoli and (B) pepper. The parameter estimates describe the effects of the ^13^C depletion of adaxial to abaxial wax ($\delta_{\text{ab}}-\delta_{\text{ad}}, ‰$) and the CO_2_ gradient across the leaf [($c_{\text{ad}}-c_{\text{ab}})/c_{\text{ab}}$] on amphistomy level (ASL). Estimates and confidence intervals (CI) are the median and 95% quantile intervals of the posterior distribution.

| 1. **Broccoli** | |  | | | |  | | |
| --- | --- | --- | --- | --- | --- | --- | --- | --- |
|  | **ASL** | | | $\boldsymbol{\delta}_{\text{ab}}\boldsymbol{-}\boldsymbol{\delta}_{\text{ad}}, ‰$ | | | $\boldsymbol{(c}_{\text{ad}}\boldsymbol{-}\boldsymbol{c}_{\text{ab}}\boldsymbol{)/}\boldsymbol{c}_{\text{ab}}$ | |
| *Predictors* | *Estimates* | | *CI (95%)* | *Estimates* | *CI (95%)* | | *Estimates* | *CI (95%)* |
| Intercept  Leaf age: young  Light treatment: low | 0.44 | | 0.41 – 0.47 | 0.41 | 0.19 – 0.99 | | 0.02 | -0.02 – 0.06 |
| Leaf age: mature | -0.04 | | -0.06 – -0.02 | 0.03 | -0.40 – 0.47 | | -0.00 | -0.02 – 0.02 |
| Leaf age: old | -0.07 | | -0.09 – -0.05 | 0.27 | -0.15 – 0.68 | | 0.01 | -0.01 – 0.03 |
| Light treatment: high | 0.03 | | 0.02 – 0.05 | 0.22 | -0.11 – 0.58 | | 0.02 | -0.00 – 0.03 |
| $\boldsymbol{\delta}_{\text{ab}}\boldsymbol{-}\boldsymbol{\delta}_{\text{ad}}, ‰$ | 0.00 | | -0.02 – 0.01 |  |  | |  |  |
| $\boldsymbol{(c}_{\text{ad}}\boldsymbol{-}\boldsymbol{c}_{\text{ab}}\boldsymbol{)/}\boldsymbol{c}_{\text{ab}}$ | -0.09 | | -0.35 – 0.16 |  |  | |  |  |
| 1. **Paprika** |  | |  |  |  | |  |  |
|  | **ASL** | | | $\boldsymbol{\delta}_{\text{ab}}\boldsymbol{-}\boldsymbol{\delta}_{\text{ad}}\boldsymbol{,}\mathbf{‰}$ | | | $\boldsymbol{(c}_{\text{ad}}\boldsymbol{-}\boldsymbol{c}_{\text{ab}}\boldsymbol{)/}\boldsymbol{c}_{\text{ab}}$ | |
| *Predictors* | *Estimates* | | *CI (95%)* | *Estimates* | *CI (95%)* | | *Estimates* | *CI (95%)* |
| Intercept  Leaf age: young  Light treatment: low | 0.13 | | 0.06 – 0.21 | 1.53 | 1.10 – 2.02 | | 0.07 | 0.04 – 0.10 |
| Leaf age: mature | 0.04 | | 0.01 – 0.07 | 0.11 | -0.16 – 0.39 | | 0.01 | -0.01 – 0.03 |
| Leaf age: old | 0.02 | | -0.01 – 0.04 | 0.33 | 0.06 – 0.60 | | 0.02 | 0.01 – 0.04 |
| Light treatment: high | 0.10 | | 0.07 – 0.13 | -0.71 | -0.94 – -0.49 | | -0.02 | -0.03 – -0.01 |
| $\boldsymbol{\delta}_{\text{ab}}\boldsymbol{-}\boldsymbol{\delta}_{\text{ad}}, ‰$ | -0.01 | | -0.04 – 0.02 |  |  | |  |  |
| $\boldsymbol{(c}_{\text{ad}}\boldsymbol{-}\boldsymbol{c}_{\text{ab}}\boldsymbol{)/}\boldsymbol{c}_{\text{ab}}$ | -0.17 | | -0.62 – 0.28 |  |  | |  |  |


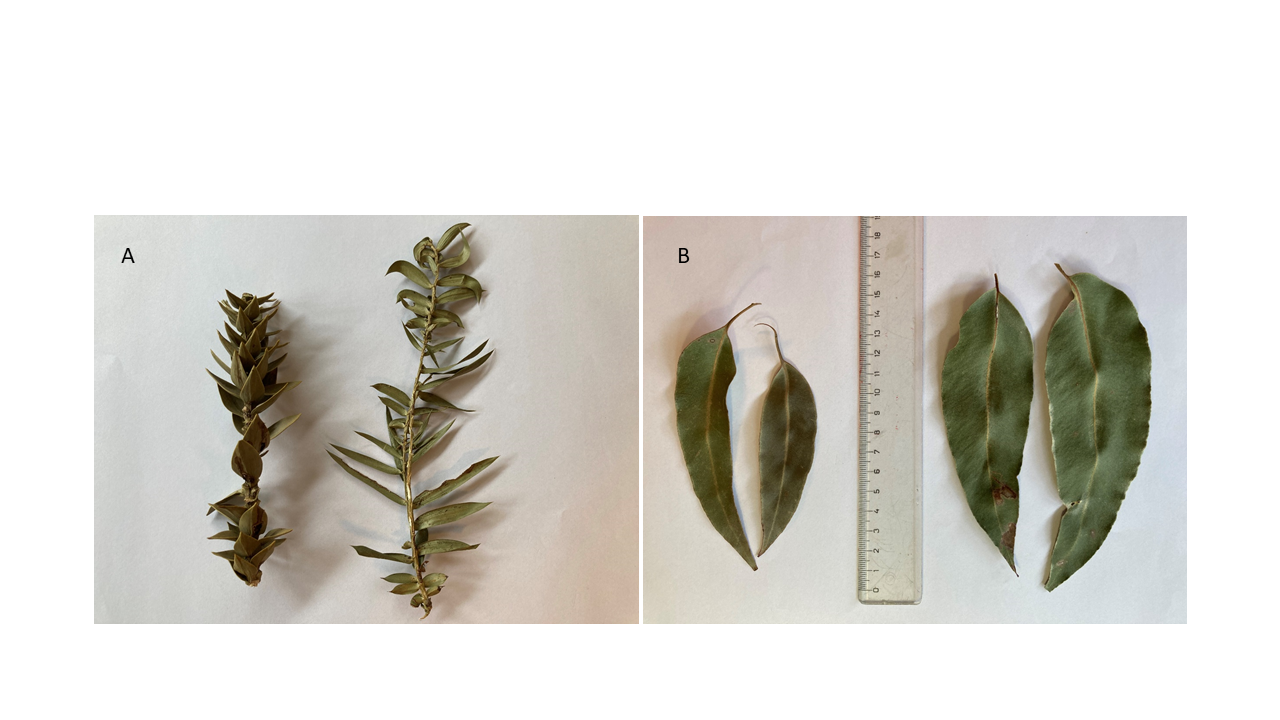


**Fig. S1**

A: Twigs with amphistomatous needles of bunya pine (*Araucaria bidwillii*) harvested from the top of crown of a mature tree (left) and from an understorey tree (right). B: Leaves of hypostomatous karri tree (*Eucalyptus diversicolor*) harvested from the top (left) and the bottom (right) of the crown of a mature tree. Leaves were dried after harvesting and epicuticular wax collected with collodion applied to both the adaxial and abaxial sides of the dry leaves.


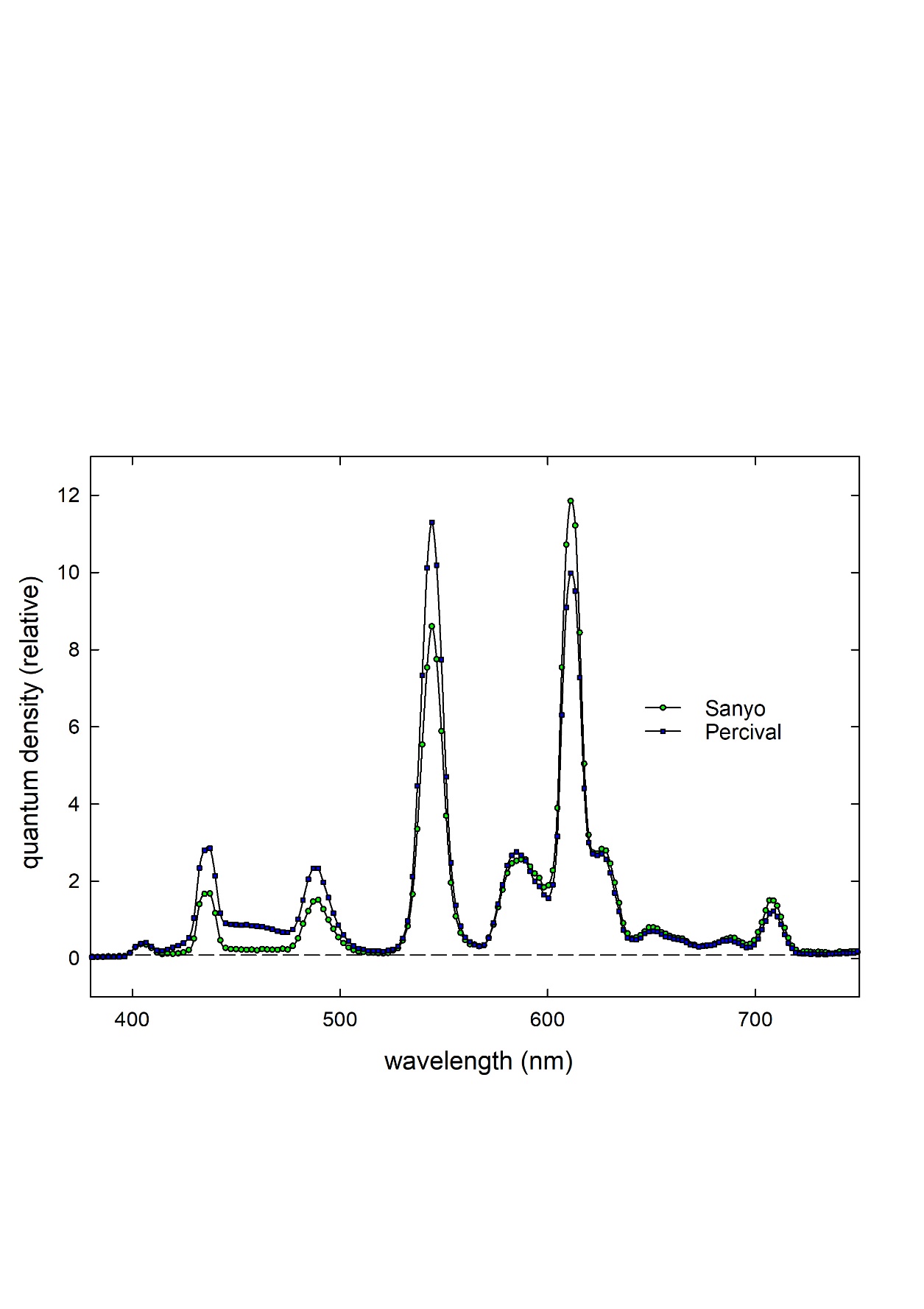


**Fig. S2**

Spectral composition of incident light used during growth of broccoli and pepper in A: Fitotron growth box (Sanyo, UK) and B: Percival growth box (Percival, Canada).


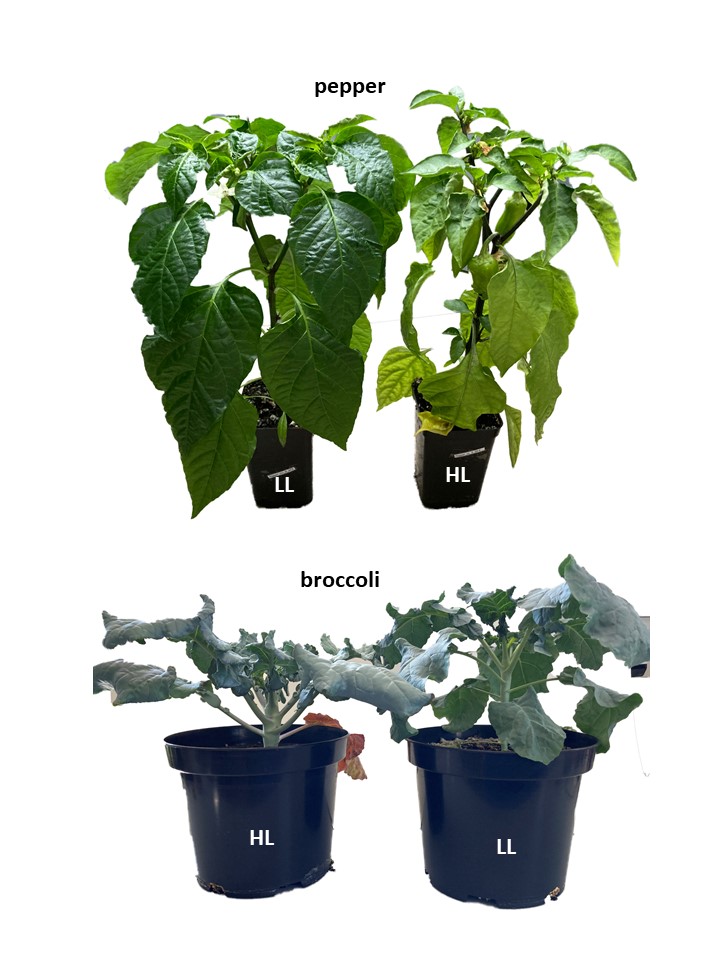


**Fig. S3**

Examples of pepper (*Capsicum annuum* L.) and broccoli (*Brassica oleracea* L., var. italica) plants grown for 13 weeks in growth box under two irradiance levels: high light, HL (450±50 μmol m^-2^ s^-1^ PPFD) and low light, LL (100±20 μmol m^-2^ s^-1^ PPFD) at day/night temperatures 25/17 °C, 16 h photoperiod.


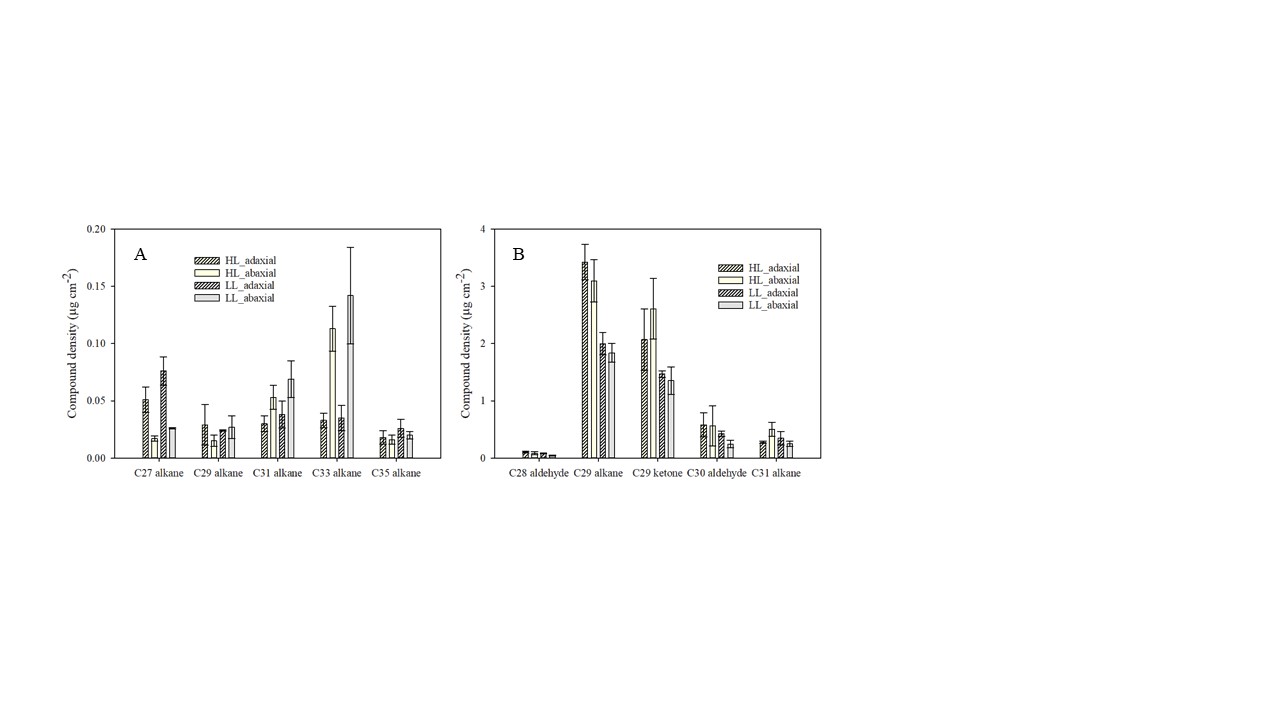


**Fig. S4**

Abundance of major compounds of epicuticular wax, EW, isolated from leaf surfaces of pepper (A) and broccoli (B) grown at high light (HL, 450±50 μmol m^-2^ s^-1^) and low light (LL, 100±20 μmol m^-2^ s^-1^). Means and standard deviations are shown (n=3).

The majority of pepper EW is formed by alkanes of various chain lengths with odd numbers of carbon (C27-C35), all of them present on both leaf sides but in different amounts. By contrast, broccoli EW is formed mainly by alkanes, ketones and aldehydes. The most abundant components in broccoli were nonacosane (C29 alkane) and nonacosan-15-one (C29 ketone), whereas in pepper tritriacontane and hentriacontane (C33 and C31 alkanes, respectively) dominated. Interestingly, pepper leaves grown under high light had a lower content of these compounds than the shaded leaves.

Method used for qualitative and quantitative wax analysis: Epicuticular wax was sampled using collodion as described in Zeisler and Schreiber (2016). Collodion strips with adhering EW were gently removed from adaxial and abaxial leaf sides using a fine tweezer. The strips were extracted over night in 2.5 ml chloroform containing 10µg of C24 alkane as internal standard. The chloroform volume was reduced under a gentle nitrogen stream to a final volume of 200µl. The polar hydroxyl- and carboxyl groups of the waxes were derivatized adding 20µl BSTFA (N,O-bis(trimethylsilyl)-trifluoroacetamid, Machery-Nagel) and 20µl pyridine (Sigma Aldrich) at 70°C for 45 min to each sample. 1 µl of each sample was analysed by on-column injection (30 m DB-1 i.d. 0.32 mm, film 0.2 µm; J&W Scientific) to a gas chromatograph coupled to a flame ionization detector (GC-FID; CGHewlett Packard 5890 series H). Wax amounts were calculated based on the amount of the internal standard used and expressed as mass of the compound per unit of leaf surface area. Identification of single wax molecules was achieved by on-column injection of 1 µl of the samples on a gas chromatograph coupled to a mass spectrometer (GC–MS; quadrupole mass selective detector HP 5971, Hewlett-Packard) and by comparing the obtained mass fragments with mass fragments described in the literature and with mass fragments stored in a homemade data library.


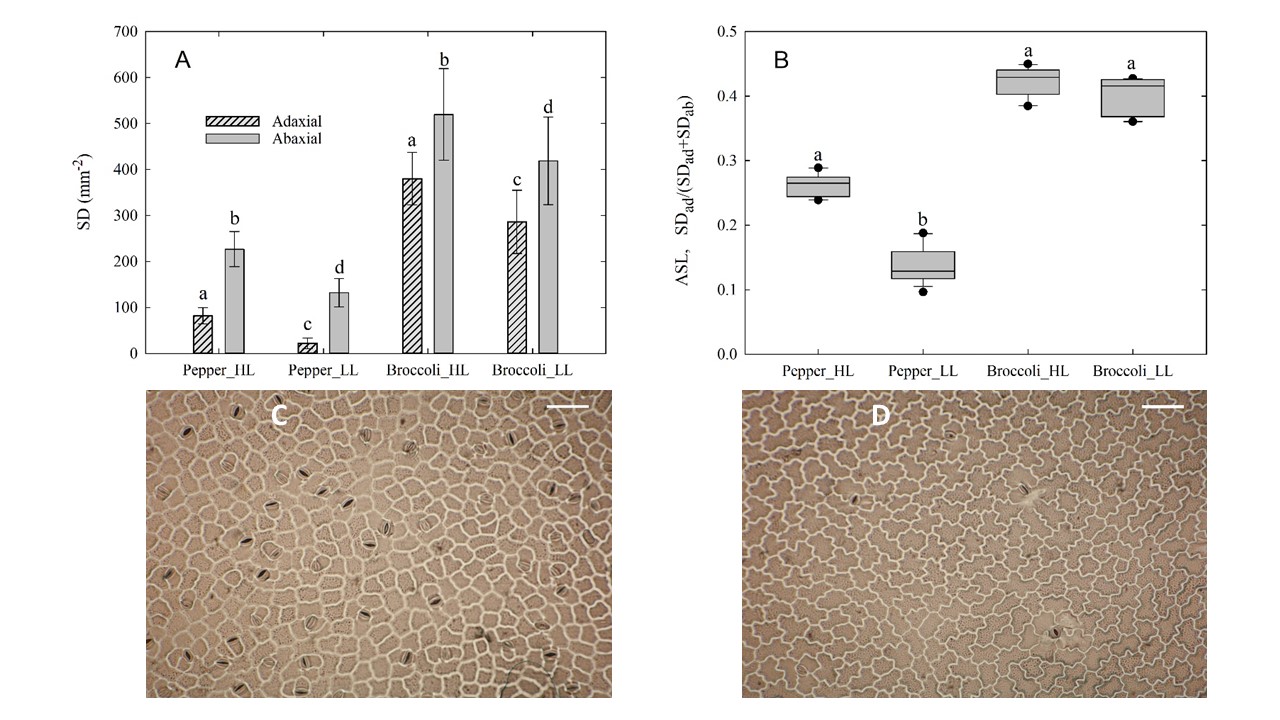


**Fig. S5**

Stomatal density and amphistomy level of pepper and broccoli leaves grown at HL (450±50 μmol m^-2^ s^-1^) and LL (100±20 μmol m^-2^ s^-1^). Data from four cultivation runs were pooled. A: Stomatal density SD. Columns and bars show mean values and standard deviation (n ≥ 10), respectively. B: Amphistomy level ASL. 25th and 75th percentiles and median are shown within the box. Whiskers (error bars) above and below the box indicate the 10-90 percentile (n ≥ 10). Identical letters above the boxes indicate the absence of statistically significant differences (one-way Anova test, P < 0.05). Light micrographs of adaxial epidermis of pepper leaf grown at HL (C) or LL (D); leaf area = 0.817 mm^2^. Bars = 0.1 mm.


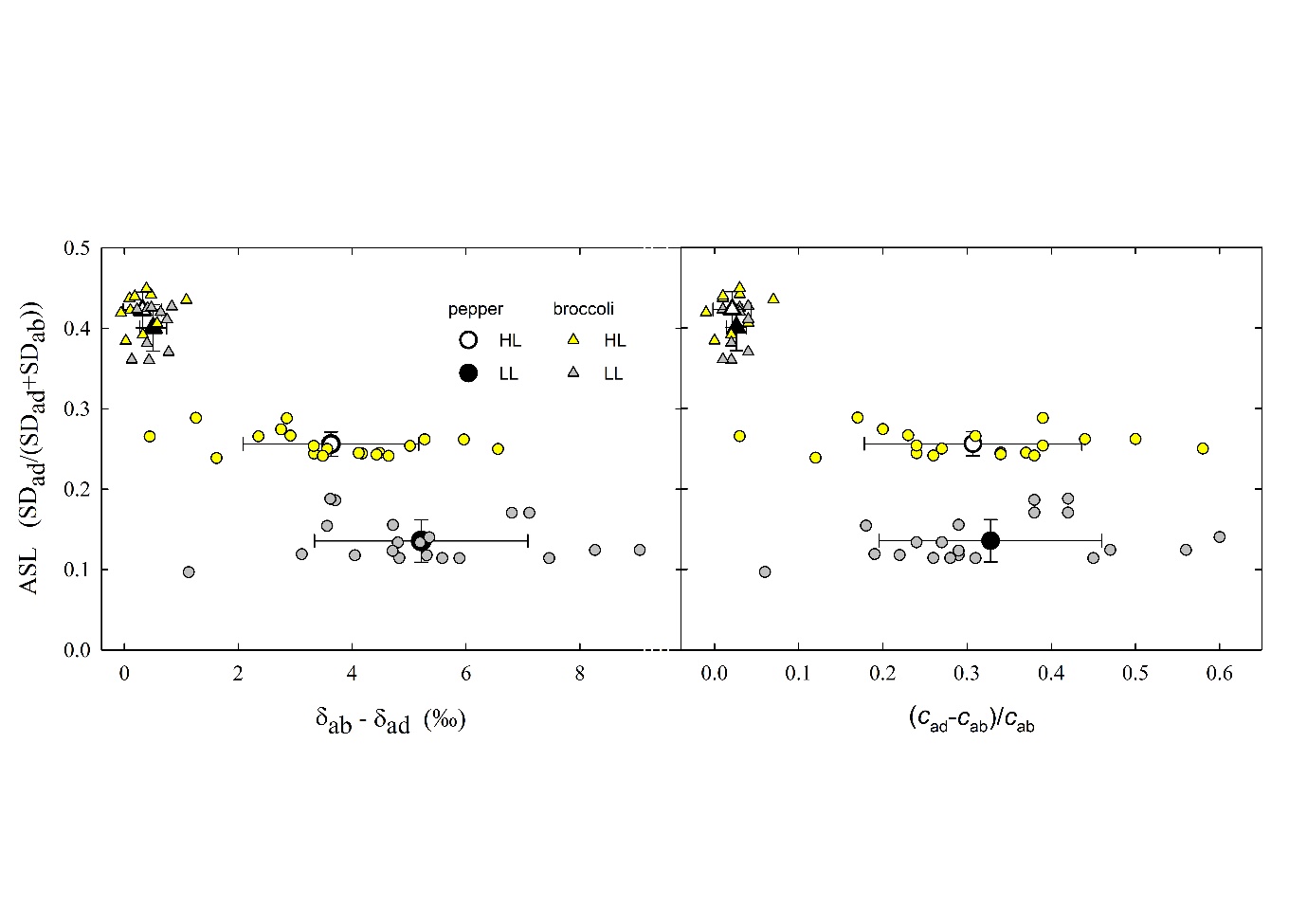


**Fig. S6**

The differences in isotopic composition between abaxial and adaxial epicuticular waxes (δ_ab_-δ_ad_) (A) and in leaf internal CO_2_ concentration across the leaf (*c*_ad_-*c*_ab_)/c_ab_ (B) plotted against amphistomy level (ASL).

Bulk epicuticular wax was used for IRMS determination of δ_ab_, δ_ad_ and bulk leaf dry mass in δ_DM_ estimation. The relative gradient (drawdown) of CO_2_ concentration across the leaf, (*c*_ad_-*c*_ab_)/c_ab_ was calculated using (δ_ab_-δ_ad_), δ_DM_ and Eq. 1 shown in Material and Methods. Points indicate values of individual plants from four experimental runs. The (δ_ab_-δ_ad_) values varied significantly between the light environments in pepper (t=2.923, P=0.006) but were much less light-dependent in broccoli (t=1.429, P=0.17). In both species more light resulted in less ^13^C depletion of adaxial compared to abaxial EW, i.e. in reduced values of δ_ab_-δ_ad_. Therefore, increasing the number of stomata on the adaxial compared to the abaxial side concomitantly decreased discrimination against ^13^C in the adaxial compared to the abaxial EW (A). The (δ_ab_-δ_ad_) values greater than zero indicate that pepper and broccoli leaves from both light environments had higher leaf internal CO_2_ concentrations near to the adaxial side. Means over the whole set of measurements and standard deviations are shown.

**
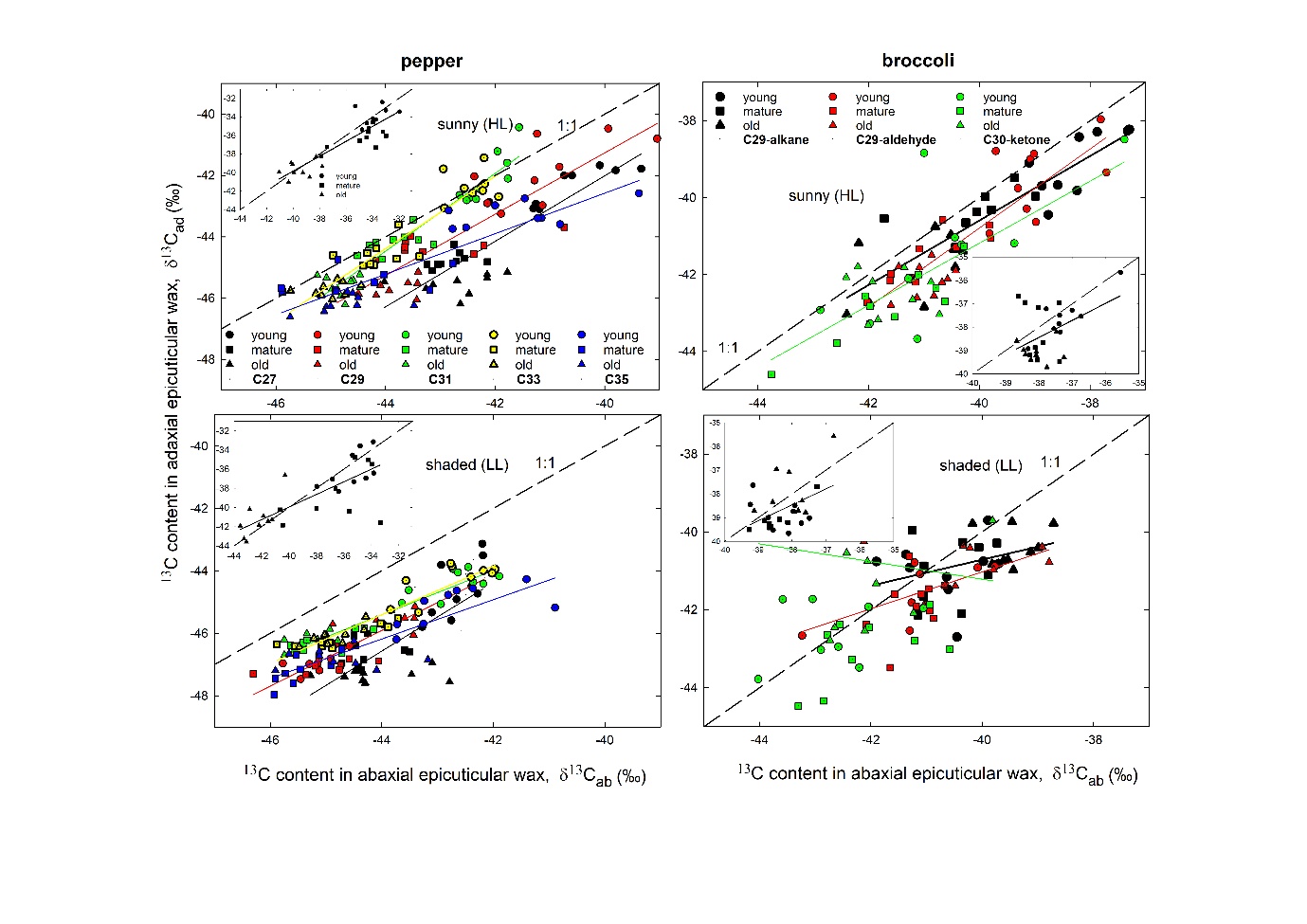
**

**Fig. S7**

Carbon isotope ratio (δ^13^C) of adaxial epicuticular wax (EW) as related to δ^13^C of abaxial EW in pepper and broccoli.

Five alkanes in pepper (C27-C35) and three aliphatics in broccoli epicuticular wax are distinguished by different colours, three leaf insertion levels by different shape of the symbols. The colour of regression lines correspond to the colour of particular compound. The insets show the same relationship estimated from the bulk wax IRMS analyses.

**
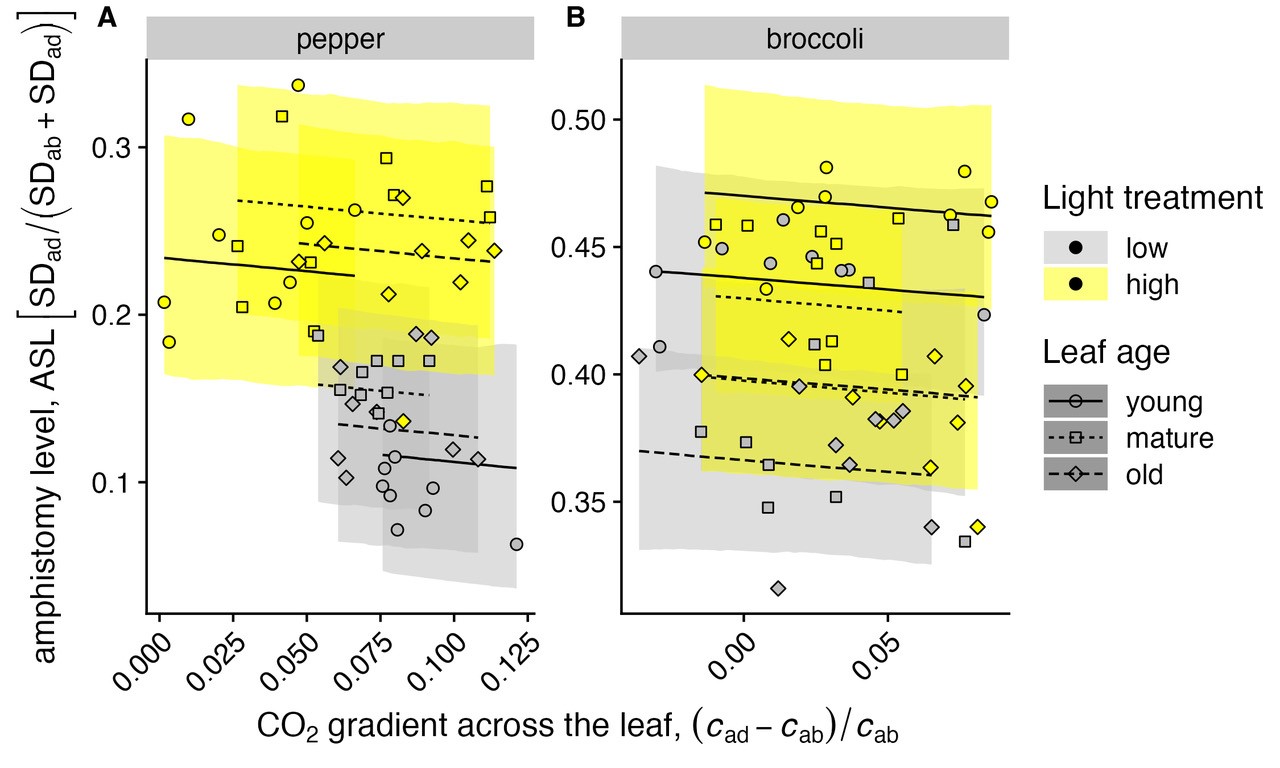
**

**Fig. S8**

Relationships between amphistomy level (ASL) of pepper (A) and broccoli (B) leaves and the relative drawdown of CO_2_ concentrations across the leaf [(*c*_ad_-*c*_ab_)/*c*_ab_] calculated using Eq. 1. Plants were grown at two different light intensities (HL, 450±50 μmol m^-2^ s^-1^, yellow symbols; LL, 100±20 μmol m^-2^ s^-1^, grey symbols); leaves of three insertion levels (ages) were analysed: low (old), middle (mature) and upper (young). Points indicate relative differences in CO_2_ concentration between the leaf sides. Lines are linear regressions of AS level on (*c*_ad_-*c*_ab_)/*c*_ab_; ribbons are 95% confident bands of the regression.

**
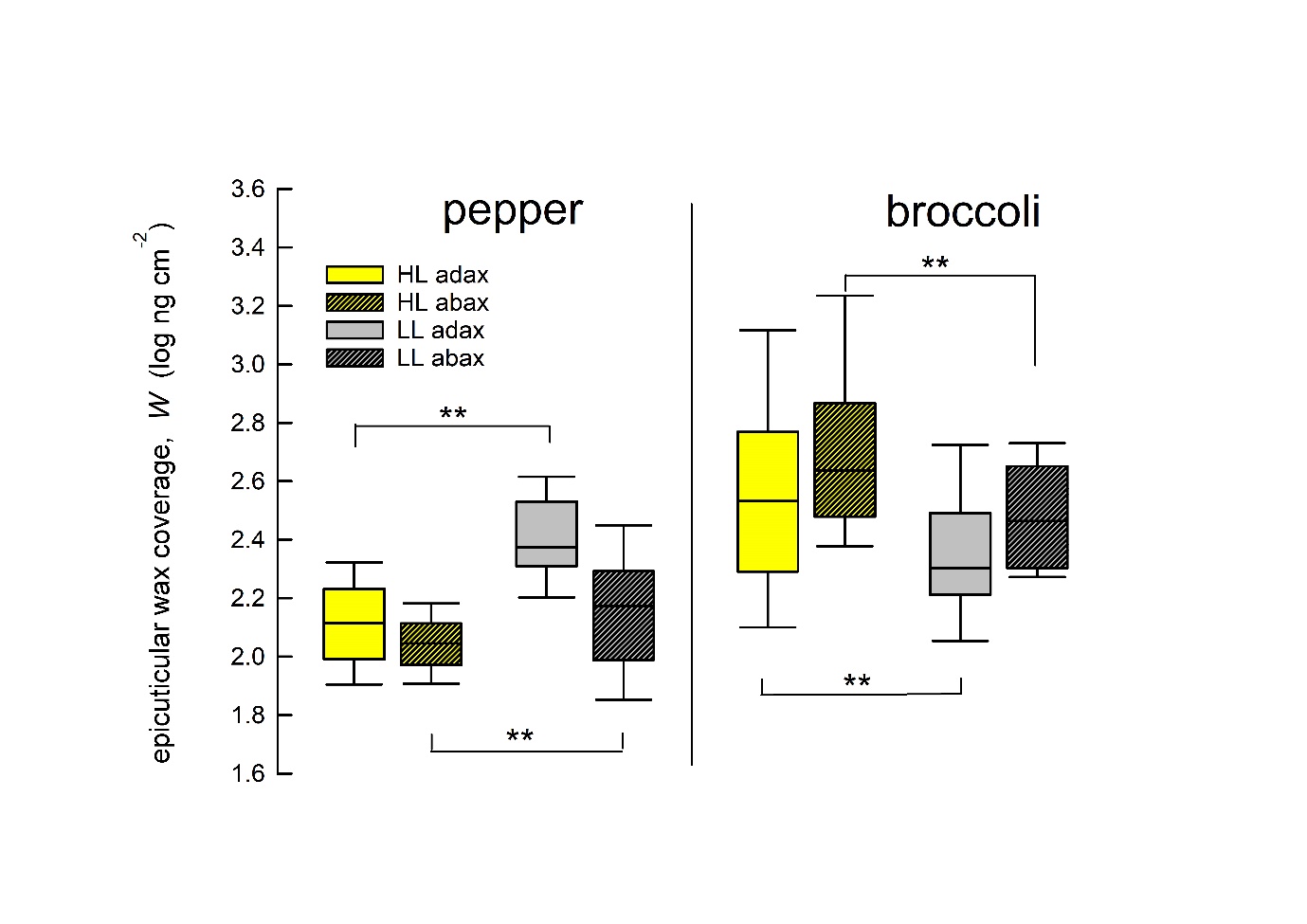
**

**Fig. S9**

Epicuticular wax coverage on adaxial and abaxial leaf sides of pepper and broccoli grown at two different irradiances.

Each box represents sum of individual wax compounds coverage weighted by their relative abundance (five odd C27-C35 alkanes in pepper and three C29-C31 aliphatic compounds in broccoli) and averaged over 27 leaves (9 from each category of young, mature and old leaves). The horizontal line inside the box is median, the lower and upper boundaries of boxes and error bars show 25-75 percentile and 10-90 percentile, respectively. Statistically significant differences between the growth light treatments (high light, HL, 450±50 μmol m^-2^ s^-1^ PPFD, and low light, LL, 100±20 μmol m^-2^ s^-1^ PPFD) are shown (*n*=27, ** indicate P<0.01).

**
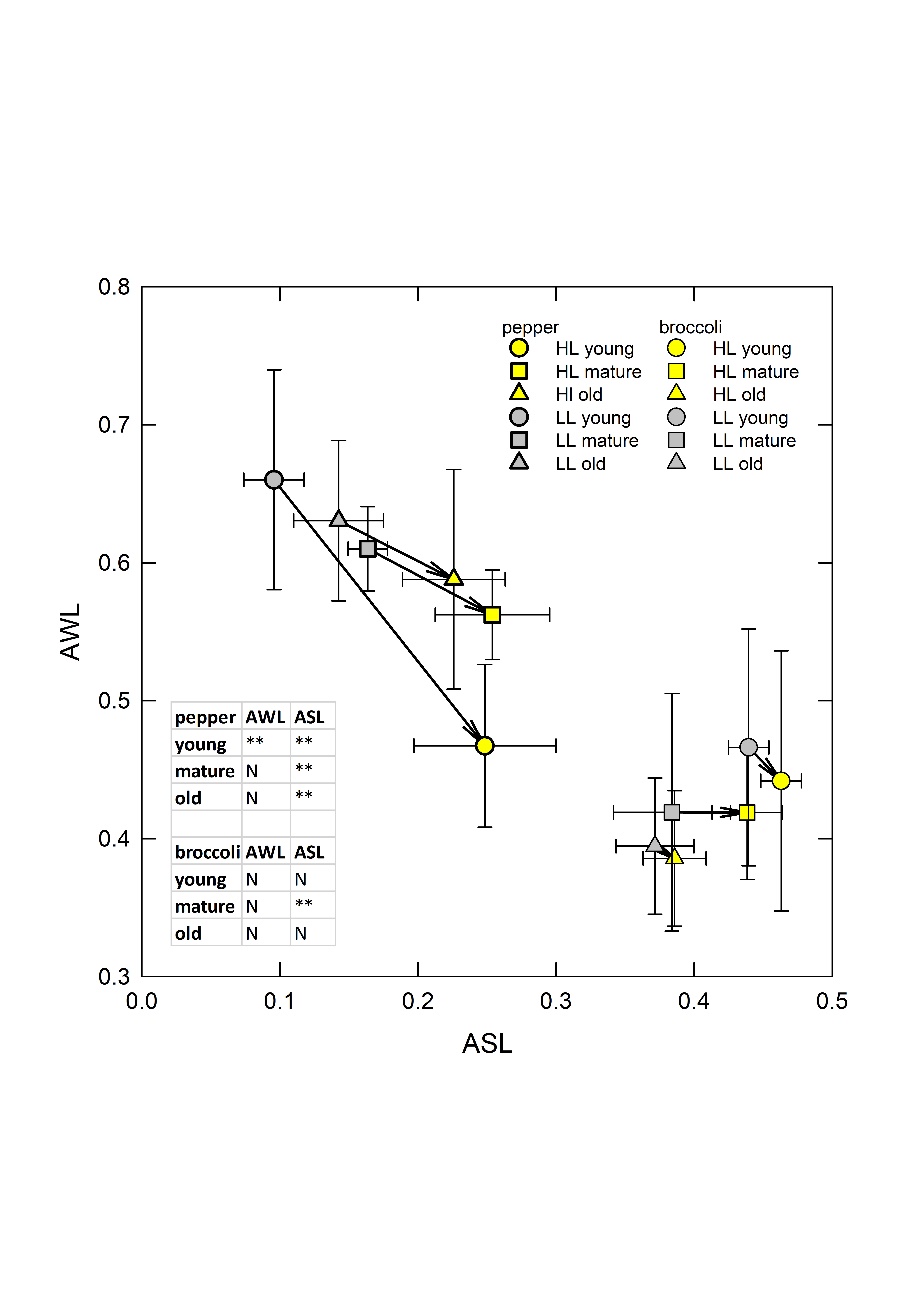
**

**Fig. S10**

The effect of light on amphistomy level (ASL) and amphiwaxy level (AWL) in pepper and broccoli leaves at three ontogeny stages.

Leaves of shaded plants (LL) have lower relative abundance of stomata on their upper leaf sides, amphistomy level [ASL =SD_ad_/(SD_ab_+SD_ad_) where SD denotes stomatal density] compared to sunny leaves (HL) of the same age. This effect of increased leaf irradiance is accompanied by reduction in the relative deposition of wax on the upper leaf side, amphiwaxy level [AWL = *W*_ad_/(*W*_ab_+*W*_ad_), where *W* is wax mass per unit area of the leaf surface]. The negative proportion between ASL and AWL driven by light as indicated by arrows is highest in the young and lowest in the old leaves in both species. The effect of light is pronounced less in broccoli than in pepper (see the inset table). Symbols represent means of nine measurements on three different plants, the bars show standard deviations.

**
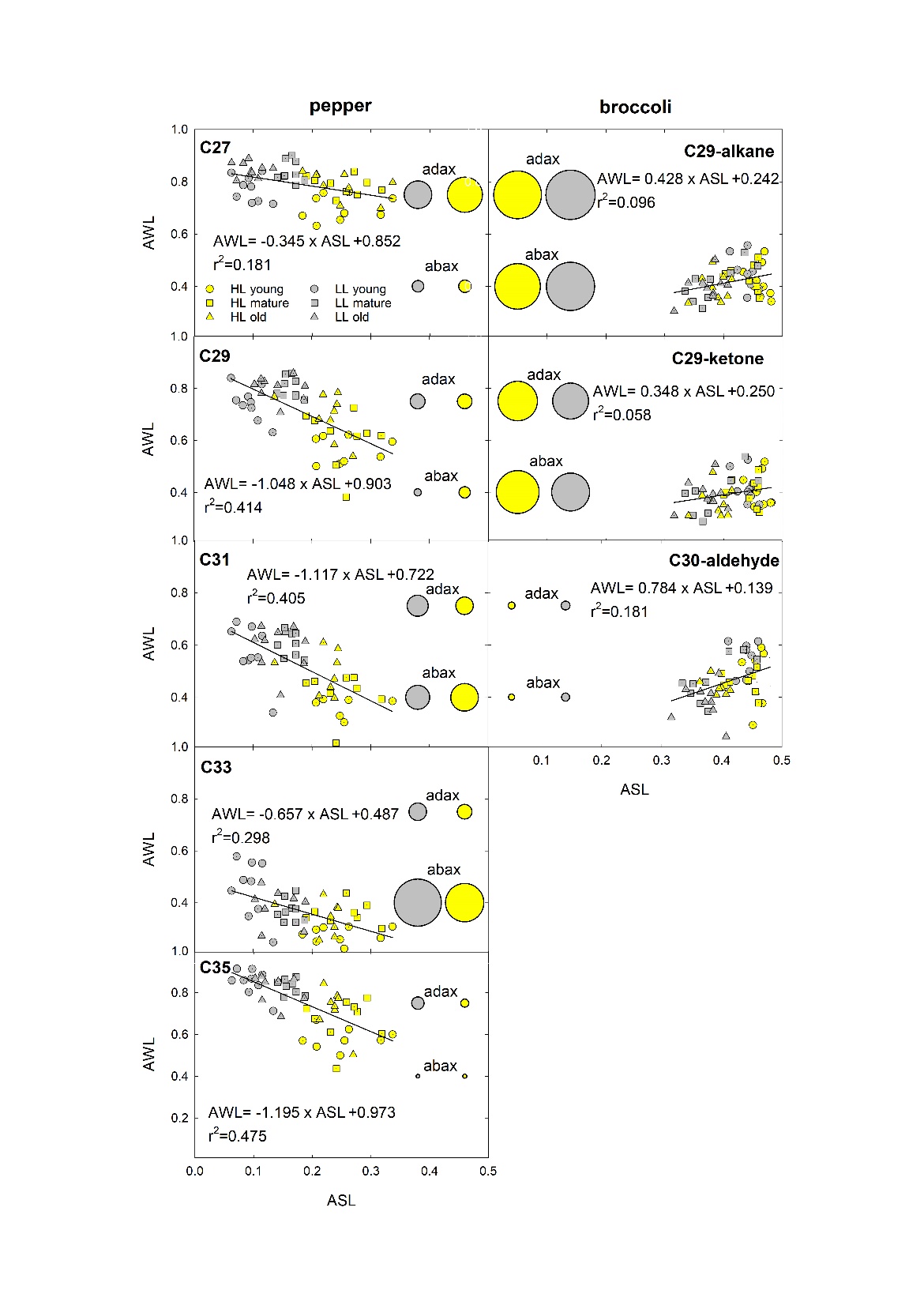
**

**Fig. S11**

Effect of light on relation of amphistomy and amphiwaxy level for major epicuticular wax compounds, five alkanes in pepper and three aliphatics in broccoli.

Bubles indicate relative abundance of the compound on adaxial and abaxial leaf sides at two growth irradiances, high light HL (yellow) and low light LL (grey). Each point represents individual leaf the age of which is distinguished by shape of the symbol.


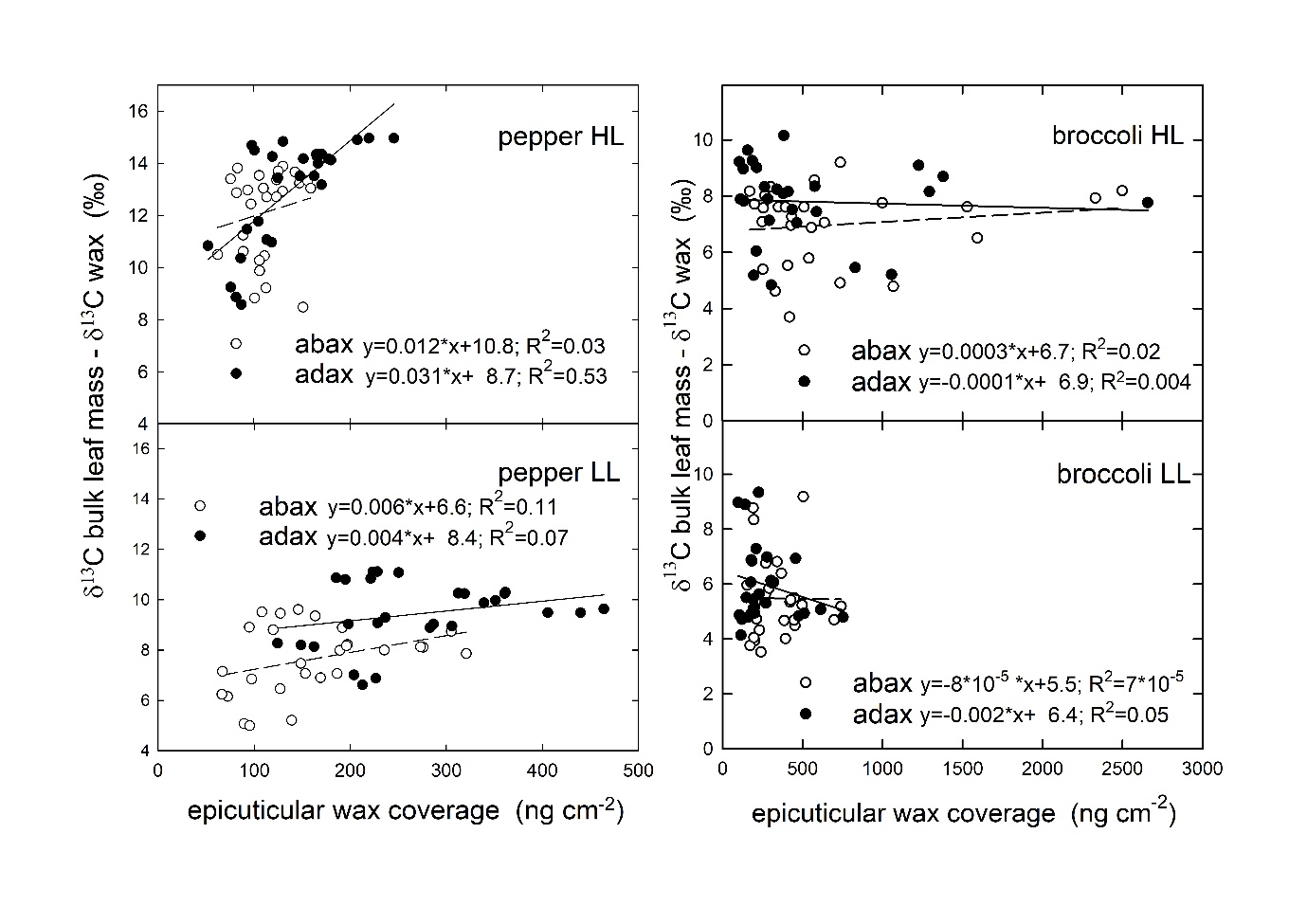


**Fig. S12**

Relationship between the depletion of epicuticular wax in ^13^C against leaf dry mass (δ_DM_-δ_wax_) and the wax coverage.

Figures indicate that the wax depletion is not related significantly to the amount of wax depositied on the adaxial and abaxial leaf surfaces of pepper and broccoli.

**
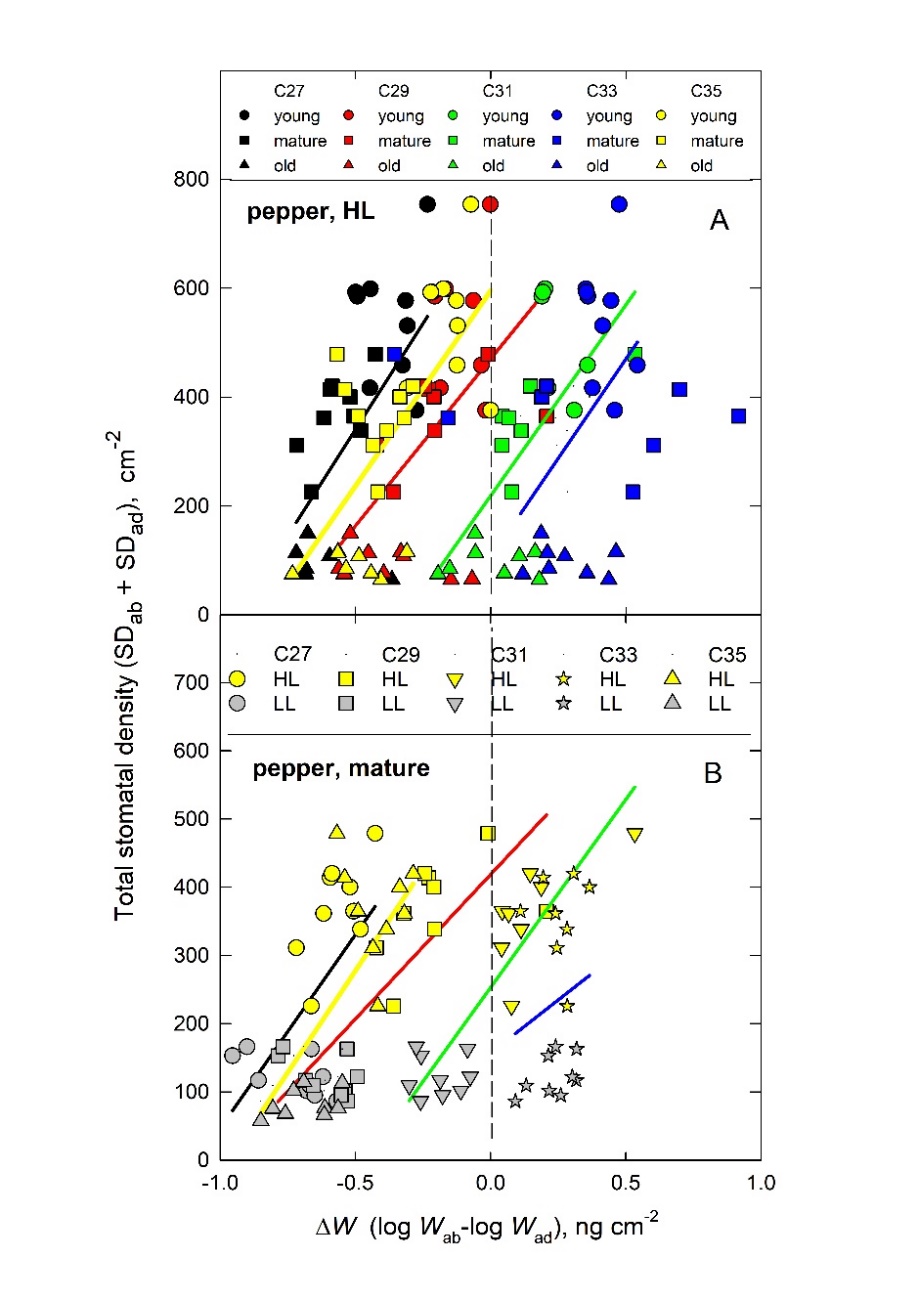
**

**Fig. S13**

Relationship between stomatal density and partitioning of epicuticular wax between the opposite sides of pepper leaf.

Leaf side-specific partitioning of five alkanes (C27-C35) correlates linearly with total number (density SD) of stomata on both leaf surfaces no matter whether the insertion level (leaf age in A) or the growth irradiance (low light LL, high light, HL in B) underlain the SD changes. The negative values of the difference in abaxial minus adaxial wax coverage, Δ*W*, indicate that epicuticular wax (EW) deposits preferentially on the adaxial leaf side, Δ*W*>0 shows preferential deposition on the abaxial side and Δ*W*=0 equal coverage on both leaf sides. With more stomata developing on the leaf, all alkanes of EW shift in their deposition toward the abaxial leaf side. Old (A) and shaded (B) leaves with the lowest number of stomata cummulate EW preferentially on adaxial leaf side.

**DeNiro MJ, Epstein S.** **1977**. Mechanism of carbon isotope fractionation associated with lipid synthesis. *Science,* **197**: 261-263.

**Haworth M, Scutt CP, Douthe C, Marino G, Gomes MTG, Loreto F, Flexas J, Centritto M.** **2018**. Allocation of the epidermis to stomata relates to stomatal physiological control: Stomatal factors involved in the evolutionary diversification of the angiosperms and development of amphistomaty. *Environmental and Experimental Botany,* **151**: 55-63.

**Park R, Epstein S.** **1961**. Metabolic fractionation of C13 and C12 in plants. *Plant Physiology,* **36**: 133-138.

**Way DA, Pearcy RW.** **2012**. Sunflecks in trees and forests: from photosynthetic physiology to global change biology. *Tree Physiology,* **32**: 1066-1081.

**Wong SC, Cowan IR, Farquhar GD.** **1985**. Leaf conductance in relation to rate of CO2 assimilation. 3. influences of water-stress and photoinhibition. *Plant Physiology,* **78**: 830-834.

**Zeisler V, Schreiber L.** **2016**. Epicuticular wax on cherry laurel (Prunus laurocerasus) leaves does not constitute the cuticular transpiration barrier. *Planta,* **243**: 65-81.

**Zhou YP, Stuart-Williams H, Grice K, Kayler ZE, Zavadlav S, Vogts A, Rommerskirchen F, Farquhar GD, Gessler A.** **2015**. Allocate carbon for a reason: Priorities are reflected in the C-13/C-12 ratios of plant lipids synthesized via three independent biosynthetic pathways. *Phytochemistry,* **111**: 14-20.
